# Supplementary material for: Exploring the impact of opioid use on outcomes in allogeneic hematopoietic stem cell transplantation
Source: PLoS One. 2025 Apr 4;20(4):e0321073. doi: 10.1371/journal.pone.0321073 (PMC11970678; doi:10.1371/journal.pone.0321073)
Supplement: S1 File — (DOCX) [file pone.0321073.s001.docx]

**Supplementary Information:**

**Inclusion/exclusion**

1207 patients transplanted between Jan 7 2010 and Dec 31 2019

After deleting second transplanted patients (n=83), we are left with n=1124 patients who were first transplanted.

After removing patients with linkable ID to opioid database (n=7), we are left with n=1117 patients.

After removing patients with incorrect data – length of follow up < 0 (n=1), we are left with n=1116 patients.

We further removed n=132 patients with the following diagnosis: Myeloma, DCN, GATA2 deficiency, MF, MPN, Non-Malign, SAA, We are left with n=984 patients.

**Definition of Opioid user and Non-opioid user**

**opioid user (n=730)**

1. Patients who started opioid use within one year after BMT, had opioid use durations* > 30 dayswithin one year after BMT (duration*: sum of durations within one year)

2. Patients who started opioid use before BMT or within one year after BMT, had opioid use durations < 30 days within one year after BMT, and **died** within one year while on opioid (n=21)

**Comparator (n=51)**

1. Patients who didn’t use opioid at all

2. Patients started opioid either before BMT or within one year post BMT, who had opioid use within one year after BMT, but total durations within 1yr < 30 days, they didn't die within 1 year while on opioid

3. Patients who started before BMT and stopped opioid before BMT

Note: total is 781.

This number is not 984, this is because patients who started after 1 year post BMT are not included, they do not qualify as opioid user or comparator (n=203)

**Sample for the analyses**

From the 781 patients, we removed 100 non-opioid users who died or last follow up occur before “median start time of opioid in the opioid group (95 days)”. We are left with n=681 patients.

cGVHD or GRFS analyses: Further removed patients died or relapsed within the first 100 days. Sample size is 514

**Start time for the analyses**

Opioid user

1. Patients who started opioid use within one year after BMT, had opioid use durations* > 30 dayswithin one year after BMT (duration*: sum of durations within one year)

**Start time = first time used opioid**

2. Patients who started opioid use before BMT or within one year after BMT, had opioid use durations < 30 days within one year after BMT, and **died** within one year while on opioid

If they started before BMT: **Start time = time of BMT**

If they started within 1 year post BMT: **Start time = first time used opioid**

Note: for these users, median start time is 95 days.

Comparator

3. Patients who didn’t use opioid at all **Start time = time of BMT + 95 days (see above)**

4. Patients started opioid either before BMT or within one year post BMT, who had opioid use within one year after BMT, but total durations within 1yr < 30 days, they didn't die within 1 year while on opioid

If they started before BMT **Start time = time of BMT + 95 days (see above)**

If they started after BMT: **Start time = opioid start time + 95 days (see above)**

4. Patients who started before BMT and stopped opioid before BMT

**Start time = time of BMT + 95 days (see above)**

**SAS Code:**

**data** data.master2;

set data.master;

/* include patients transplanted from Jan 1 2010 - Dec 31 2020

checked: already satisfy this requirement. */

/* cohort censoring date */

cohort_censor=mdy(**12**,**31**,**2021**);

/* delete second tx patients. because their length of follow up is recorded post second tx */

if days_to_date_of_2nd_bmt>**0** or _2nd_transplant_indication ne "" or date_of_2nd_bmt_0001 ne "" then delete;

/* delete the 9 patients without "encrypted ICES key number" */

if study_ID="" then delete;

/* delete the 1 patient whose length of follow up is <0 */

if study_id="xxxx" then delete;

**run**;

**data** data.master3;

set master2;

if gvh_prophylaxis in ("ATG (7.5 mg/kg)-CSA-cellcept","ATG, CSA, MTX","ATG, PTCy, CSA","ATG,CSA, MTX","ATG-CSA-MMF","ATG-CSA-MTX","ATG-MTX-CSA","ATG-PTCy-CSA",

"ATG-PtCy-CSA","CSA, MF, ATG","CSA, MF,ATG","CSA,MF,ATG","PTCy-CSA","PTCy-CSA-MMF")

then GVHD_Proph_rev="ATG-CSA-X. or PtCy-CSA.X";

else if gvh_prophylaxis="" then GVHD_Proph_rev="";

else GVHD_Proph_rev="Others";

if agvh_grade in ("grade1","grade2","grade3","grade4") then AGVH_grade_rev=AGVH_grade;

else if agvh_grade in ("N/A","") then AGVH_grade_rev="";

if AGVH_grade_rev in ("grade3","grade4") then AGVH_grade_rev2="Grade 3 - 4";

else if AGVH_grade_rev in ("grade1","grade2") then AGVH_grade_rev2="Grade 1 - 2";

else AGVH_grade_rev2="";

if cgvh_grade in ("Moderate to Severe","Moderate to severe","Severe","grade2","moderate","moderate to severe","severe","severe (autopsy)")

then cgvh_grade_rev="Moderate to Severe";

else if cgvh_grade="Mild" then cgvh_grade_rev="Mild";

else if cgvh_grade in ("cgvh_grade","") then cgvh_grade_rev="";

cd34_dose_num=input(cd34_dose, best12.);

day_30_survival_num=input(day_30_survival, best12.);

day_100_survival_num=input(day_100_survival, best12.);

day_365_survival_num=input(day_365_survival, best12.);

if vod ne "" then vod_ind=**1**; else vod_ind=**0**;

if toxicity ne "" or other_toxicity ne "" or other_toxicity_0001 ne "" or other_toxicity_0002 ne "" or other_toxicity_0003 ne ""

then toxicity_ind=**1**; else toxicity_ind=**0**;

if pneumonia ne "" or pneumonia_0001 ne "" or pneumonia_0002 ne "" then pneumonia_ind=**1**; else pneumonia_ind=**0**;

length neut_recovery_rev $100.;

if neut_recovery="Never recovered" then neut_recovery_rev="";

else if neut_recovery="D+8" then neut_recovery_rev="D+08";

else if neut_recovery="D+9" then neut_recovery_rev="D+09";

else neut_recovery_rev=neut_recovery;

neut_recovery_num= input(substr(neut_recovery_rev,**3**,**2**),best12.) ;

length plt_recovery_20_cibmtr_rev $100.;

if plt_recovery__20_cibmtr in ("Never Recovered","Never below 20","Never recovered","never recovered") then plt_recovery_20_cibmtr_rev="";

else plt_recovery_20_cibmtr_rev=substr(plt_recovery__20_cibmtr, **3**);

plt_recovery_20_cibmtr_num=input(plt_recovery_20_cibmtr_rev, best12.);

length plt_recovery__20_rev $100.;

if plt_recovery__20 in ("Never below 20","Never recovered","never recovered") then plt_recovery__20_rev="";

else plt_recovery__20_rev=substr(plt_recovery__20, **3**);

plt_recovery_20_num=input(plt_recovery__20_rev, best12.);

length plt__30_rev $100.;

if plt__30 in ("Never above 30","Never below 30","Never recovered") then plt__30_rev="";

else plt__30_rev=substr(plt__30, **3**);

plt_recovery_30_num=input(plt__30_rev, best12.);

if days_to_date_of_death ne **.** then dead_indicator=**1**; else dead_indicator=**0**;

if days_to_agvhd_date ne **.** then aGVHD_indicator=**1**; else aGVHD_indicator=**0**;

if days_to_bsi_date ne **.** then BSI_indicator=**1**;else BSI_indicator=**0**;

if days_to_cgvhd_date ne **.** then cGVHD_indicator=**1**;else cGVHD_indicator=**0**;

if days_to_cmv__1 ne **.** then CMV_indicator=**1**; else CMV_indicator=**0**;

if days_to_date_of_toxicity ne **.** then toxicity_indicator=**1**; else toxicity_indicator=**0**;

if days_to_ebv_reactivation ne **.** then EBV_indicator=**1**; else EBV_indicator=**0**;

if days_to_max_ebv_date ne **.** then Max_EBV_indicator=**1**; else Max_EBV_indicator=**0**;

if days_to_graft_failure ne **.** then GF_indicator=**1**; else GF_indicator=**0**;

if days_to_ifi_date ne **.** then IFI_indicator=**1**; else IFI_indicator=**0**;

if days_to_pneumonia_date ne **.** then Pneumonina_indicator=**1**; else Pneumonina_indicator=**0**;

if days_to_relapse_date ne **.** then relapse_indicator=**1**; else relapse_indicator=**0**;

if days_to_vod_date ne **.** then vod_indicator=**1**; else vod_indicator=**0**;

if AGVH_grade_rev2="Grade 3 - 4" then do; days_to_agvhd34_date=days_to_agvhd_date; aGVHD34_indicator=**1**; end;

else if AGVH_grade_rev2="Grade 1 - 2" then do; days_to_agvhd34_date=days_to_agvhd_date; aGVHD34_indicator=**0**; end;

if cgvh_grade_rev="Moderate to Severe" then do; days_to_cGVHD_mod_sev_date=days_to_cgvhd_date; cgvh_mod_sev_indicator=**1**; end;

else if cgvh_grade_rev="Mild" then do; days_to_cGVHD_mod_sev_date=days_to_cgvhd_date; cgvh_mod_sev_indicator=**0**; end;

**run**;

%***table1macro***(dsn=master3,

calist= /* age at BMT */ Age_Group sex

/* nearest census based neighbourhood income quintile (within CMA/CA) */ incquint /* 2008 rurality index for ontario */rio2008

/* charlson index */ charl dependency_q_da deprivation_q_da ethnicdiv_q_da ethnicdiv_q_da

diagnose donor hla_match stage donor_gender source frozen_fresh ric_mac GVHD_Proph_rev recipient_cmv donor_cmv

recipient_abo donor_cmv donor_abo abo_compatibility

kps_prior_sct primary_disease_1_therapy_relat STAGE_AT_TRANSPLANT_ANY_CR_1_CR kps_prior_sct

hct_ci dri_0_low___1_intermediate_2_hi primary_disease_1_therapy_relat STAGE_AT_TRANSPLANT_ANY_CR_1_CR

classification_aml_acording_to_ cr1__cr1__required_2_inductions cytogenetics___favorable_1_inte

ptld

arrhythmia cardiac inflammatory_bowel_disease diabetes cerebrovascular_disease psychiatric_disturbance hepatic__mild obesity infection rheumatologic

peptic_ulcer renal moderate_severe_renal moderate_pulmonary prior_solid_tumor heart_valve_disease severe_pulmonary moderate_severe_hepatic

vod_ind toxicity_ind agvh_grade_rev cgvh_grade_rev /* toxicity pneumonia */ EBV EBV_react /*BSI_ind pneumonia_ind */

gf relapse graft_failure_vs__relapse GF_indicator relapse_indicator

dead dead_indicator

aGVHD_indicator aGVHD34_indicator cgvh_indicator cgvh_mod_sev_indicator

BSI_indicator CMV_indicator toxicity_indicator

EBV_indicator Max_EBV_indicator IFI_indicator Pneumonina_indicator vod_indicator ,

coplist= donor_age cd34_dose_num kps_prior_sct hct_ci

day_30_chimerism day_60_chimerism day_90_chimerism day_120_chimerism day_180_chimerism day_360_chimerism kps_prior_sct hct_ci

day_30_survival_num day_100_survival_num day_365_survival_num days_to_discharge

neut_recovery_num plt_recovery_20_cibmtr_num plt_recovery_20_num plt_recovery_30_num

Length_of_follow_up days_to_end_fu days_to_last_f_u days_to_date_of_death days_to_agvhd_date days_to_bsi_date days_to_cgvhd_date days_to_cmv__1

days_to_date_of_toxicity

days_to_ebv_reactivation days_to_max_ebv_date days_to_graft_failure days_to_ifi_date days_to_pneumonia_date days_to_relapse_date days_to_vod_date

days_to_agvhd34_date days_to_cGVHD_mod_sev_date,

cononplist= donor_age cd34_dose_num kps_prior_sct hct_ci

day_30_chimerism day_60_chimerism day_90_chimerism day_120_chimerism day_180_chimerism day_360_chimerism kps_prior_sct hct_ci

day_30_survival_num day_100_survival_num day_365_survival_num days_to_discharge

neut_recovery_num plt_recovery_20_cibmtr_num plt_recovery_20_num plt_recovery_30_num

Length_of_follow_up days_to_end_fu days_to_last_f_u days_to_date_of_death days_to_agvhd_date days_to_bsi_date days_to_cgvhd_date days_to_cmv__1

days_to_date_of_toxicity

days_to_ebv_reactivation days_to_max_ebv_date days_to_graft_failure days_to_ifi_date days_to_pneumonia_date days_to_relapse_date days_to_vod_date

days_to_agvhd34_date days_to_cGVHD_mod_sev_date,

caincludemissing=yes,

copincludemissing=yes,

cononpincludemissing=yes,

overall=yes,

cononpsupplement=range,

missingtop=no,

group= );

/* remove non opioid related drugs */

**data** opioid;

set data.nms;

if din_desc in ("Act Amphetamine XR","Adderall XR","Androderm","Androgel","Apo-Alpraz","Apo-Bromazepam","Apo-Clobazam","Apo-Clonazepam","Apo-Dextroamphetamine",

"Apo-Diazepam","Apo-Flurazepam","Apo-Lorazepam","Apo-Lorazepam Sublingual","Apo-Methylphenidate","Apo-Oxazepam","Apo-Oxycodone CR","Apo-Temazepam",

"Apo-Zolpidem ODT","Atasol-30","Ativan","Ativan Sublingual","Axiron","Cassette","Cesamet","Co Nabilone","CoActifed","Concerta","Cophylac Drops",

"Delatestryl","Demerol","Depo-Testosterone","Diazepam","Dimetane Expectorant C","Dimetane Expectorant DC","Dimetapp-C Syrup","Fiorinal C1/2",

"Fiorinal C1/4","Generic Monitored Drug Compound","Hycodan","Injectable mixtures","KETALAR","Lomotil","Lomotil","Lorazepam","Lorazepam Injection USP"

"Lorazepam Sublingual",

"Meperidine","Midazolam","Midazolam Inj. SDZ (Preservative Free)","Midazolam Injection","Mogadon","Mylan-Alprazolam","Mylan-Clonazepam","Novahistex DH",

"Novahistine DH","Novo-Clobazam","Novo-Clonazepam","Novo-Lorazem","Other Infn Device up to 100mL","PMS-Clonazepam","PMS-Clonazepam Tab 1.0mg",

"PMS-Clonazepam- Tab 0.25mg","PMS-Clonazepam-R","PMS-Lorazepam","PMS-Methylphenidate","PMS-Nabilone","PMS-Testosterone","PMS-Zolpidem ODT",

"Phenobarbital Injection","Ran-Nabilone","Ratio-Cotridin","Ratio-Cotridin Expectorant","Ratio-Tecnal C1/2","Ratio-Tecnal C1/4","Restoril","Ritalin",

"Rivotril","Robitussin Ac","Sandoz Clonazepam","Sandoz Lorazepam","Sandoz Midazolam","Sandoz Nitrazepam","Sublinox","Taro-Testosterone",

"Taro-Testosterone Cypionate Injection","Taro-Testosterone Gel","Temazepam","Teva-Alprazolam","Teva-Clobazam","Teva-Lorazepam","Teva-Nabilone",

"Triazolam","Tussionex","Vyvanse","Xanax TS")

then do; strength=""; dosage_form=""; quantity=**.**; dayssupl=**.**; days_to_dt_of_serv_ts=**.**; days_to_adjudication_dt_ts=**.**; end;

**run**;

/* morphin conversion */

**data** opioid2;

set opioid;

if din_desc="Abstral" then morphine=**100**;

else if din_desc="Act Buprenorphine/Naloxone" then morphine=**80**;

else if din_desc="Apo-Fentanyl Matrix" then morphine=**100**;

else if din_desc="Apo-HYDROmorphone CR" then morphine=**5**;

else if din_desc="Apo-Hydromorphone" then morphine=**5**;

else if din_desc="Apo-Oxycodone CR" then morphine=**1.5**;

else if din_desc="Apo-Oxycodone/Acet" then morphine=**1.5**;

else if din_desc="Apo-Tramadol" then morphine=**0.1**;

else if din_desc="Apo-Tramadol/Acet" then morphine=**0.1**;

else if din_desc="Auro-Tramadol" then morphine=**0.1**;

else if din_desc="Butrans 10" then morphine=**80**;

else if din_desc="Butrans 20" then morphine=**80**;

else if din_desc="Butrans 5" then morphine=**80**;

else if din_desc="Codeine Contin" then morphine=**0.1**;

else if din_desc="Codeine Phosphate Sirop De" then morphine=**0.1**;

else if din_desc="Codeine Phosphate Syrup" then morphine=**0.1**;

else if din_desc="Dilaudid" then morphine=**5**;

else if din_desc="Doloral 1" then morphine=**1**;

else if din_desc="Doloral 5 Sirop 5mg/mL" then morphine=**1**;

else if din_desc="Durela" then morphine=**0.1**;

else if din_desc="Endocet" then morphine=**1.5**;

else if din_desc="Hydromorph Contin" then morphine=**5**;

else if din_desc="Hydromorphone" then morphine=**5**;

else if din_desc="Hydromorphone HP-10" then morphine=**5**;

else if din_desc="Jamp-Acet-Tramadol" then morphine=**0.1**;

else if din_desc="Jurnista" then morphine=**5**;

else if din_desc="Kadian" then morphine=**1**;

else if din_desc="M-Eslon" then morphine=**1**;

else if din_desc="MS IR Tab 10mg" then morphine=**1**;

else if din_desc="MS IR Tab 5mg" then morphine=**1**;

else if din_desc="Mar-Tramadol/Acet" then morphine=**0.1**;

else if din_desc="Metadol" then morphine=**10**;

else if din_desc="Methadone" then morphine=**10**;

else if din_desc="Methadose" then morphine=**10**;

else if din_desc="Mint-Tramadol/Acet" then morphine=**0.1**;

else if din_desc="Morphine HP-50" then morphine=**1**;

else if din_desc="Morphine Sulfate" then morphine=**1**;

else if din_desc="Morphine Sulfate Injection USP" then morphine=**1**;

else if din_desc="Mylan-Buprenorphine/Naloxone" then morphine=**80**;

else if din_desc="Mylan-Tramadol/Acet" then morphine=**0.1**;

else if din_desc="Novo-Morphine SR" then morphine=**1**;

else if din_desc="Nucynta CR" then morphine=**0.33**;

else if din_desc="Nucynta ER" then morphine=**0.33**;

else if din_desc="Nucynta IR" then morphine=**0.33**;

else if din_desc="Opium & Belladonna" then morphine=**0.1**;

else if din_desc="Oxy.IR" then morphine=**1.5**;

else if din_desc="OxyNEO" then morphine=**1.5**;

else if din_desc="Oxycontin" then morphine=**1.5**;

else if din_desc="PMS-Buprenorphine-Naloxone" then morphine=**80**;

else if din_desc="PMS-HYDROmorphone" then morphine=**5**;

else if din_desc="PMS-Hydrocodone" then morphine=**0.67**;

else if din_desc="PMS-Oxycodone" then morphine=**1.5**;

else if din_desc="PMS-Oxycodone CR" then morphine=**1.5**;

else if din_desc="Percocet" then morphine=**1.5**;

else if din_desc="Percocet-Demi" then morphine=**1.5**;

else if din_desc="Ralivia" then morphine=**0.1**;

else if din_desc="Ran-Fentanyl Matrix Patch" then morphine=**100**;

else if din_desc="Ran-Tramadol/Acet" then morphine=**0.1**;

else if din_desc="Ratio-Codeine" then morphine=**0.1**;

else if din_desc="Ratio-Fentanyl" then morphine=**100**;

else if din_desc="Ratio-Lenoltec No.2" then morphine=**100**;

else if din_desc="Ratio-Lenoltec No.3" then morphine=**100**;

else if din_desc="Ratio-Lenoltec No.4" then morphine=**100**;

else if din_desc="Ratio-Morphine" then morphine=**1**;

else if din_desc="Ratio-Oxycocet" then morphine=**1.5**;

else if din_desc="Sandoz Fentanyl Patch" then morphine=**100**;

else if din_desc="Sandoz Morphine SR" then morphine=**1**;

else if din_desc="Sandoz Oxycodone/Acetaminophen" then morphine=**1.5**;

else if din_desc="Statex" then morphine=**1**;

else if din_desc="Suboxone" then morphine=**80**;

else if din_desc="Supeudol" then morphine=**1.5**;

else if din_desc="Targin" then morphine=**1.5**;

else if din_desc="Taro-Tramadol ER" then morphine=**0.1**;

else if din_desc="Teva-Codeine" then morphine=**0.1**;

else if din_desc="Teva-Fentanyl" then morphine=**100**;

else if din_desc="Teva-Hydromorphone" then morphine=**5**;

else if din_desc="Teva-Lenoltec No.2" then morphine=**0.1**;

else if din_desc="Teva-Lenoltec No.3" then morphine=**0.1**;

else if din_desc="Teva-Morphine SR" then morphine=**1**;

else if din_desc="Teva-Oxycocet" then morphine=**1.5**;

else if din_desc="Teva-Tramadol/Acetaminophen" then morphine=**0.1**;

else if din_desc="Tramadol" then morphine=**0.1**;

else if din_desc="Tridural" then morphine=**0.1**;

else if din_desc="Tylenol with Codeine No. 2" then morphine=**0.1**;

else if din_desc="Tylenol with Codeine No. 3" then morphine=**0.1**;

else if din_desc="Ultram" then morphine=**0.1**;

**run**;

/***********************************************************************/

/********************* PATIENT LEVEL **********************************/

/***********************************************************************/

**proc** **sort** data=opioid2; by study_id days_to_dt_of_serv_ts; **run**;

**data** opioid5;

set opioid2;

by study_ID;

if first.study_ID then

drug_number=**0**;

drug_number+**1**;

if strength in ("100mcg/hr","10mcg/hr","12mcg/hr","25mcg/hr","50mcg/hr","75mcg/hr") then quantity_rev=**1**;

else quantity_rev=quantity;

**run**;

**data** opioid6;

set opioid5;

identifier=cats(study_ID, drug_number);

**run**;

/******** In order to definine opioid users vs non-users,

we first calculate duration of operiod use.

For records with overlaps, take the longest contiuous duration.

If there are gaps between records, keep them as seperate records ****************/

**data** clean;

set

opioid5(keep=study_id days_to_dt_of_serv_ts in=inStart rename=(days_to_dt_of_serv_ts=date))

opioid5(keep=study_id end_date in=inEnd rename=(end_date=date));

if inStart then marker=**1**;

else marker=-**1**;

**run**;

**proc** **sort** data=clean;

by study_id date;

**run**;

**data** clean2;

set clean;

by study_id;

if first.study_ID then cumulator=**0**;

cumulator+marker;

**run**;

**data** clean3;

set clean2;

by study_ID;

if first.study_ID or lag(cumulator)=**0** then

do;

retain start end_date;

start=date;

end_date=date;

end;

start = start >< date;

end_date = end_date <> date;

if cumulator=**0** then output;

keep study_ID start end_date;

**run**;

/* calculate gap */

**data** gap;

set clean3;

by study_ID;

lag_end=lag(end_date);

gap=start-lag_end;

interval_days=end_date-start;

if first.study_ID then do;

lag_end=**.**;

gap=**.**;

end;

**run**;

/* record number: assign record number to distinct drug use period. It is organized in time order */

**data** record_number;

set gap;

by study_ID;

if first.study_ID then

record_number=**0**;

record_number+**1**;

**run**;

/* cumulative days: cumulatively how much time is spent in each period */

**data** cumulative_days;

set record_number;

by study_ID;

if first.study_ID then

cummulative_days=**0**;

cummulative_days+interval_days;

keep study_id cummulative_days;

**run**;

/* total number of days */

**data** total;

set record_number2;

by study_ID;

if first.study_ID then

total_days=**0**;

total_days+interval_days;

if last.study_ID;

keep study_id total_days;

**run**;

**data** record_total;

merge record_number2 total cumulative_days;

by study_ID;

drop lag_end;

**run**;

**data** record_total_update;

set record_total;

by study_ID;

if first.study_ID;

**run**;

/***********************************************************************************/

/****************************************** Merge *********************************/

/***********************************************************************************/

**data** record_total_for_merge;set record_total; identifier=cats(study_ID, record_number); **run**;

**proc** **sort** data=record_total_for_merge; by study_ID record_number; **run**;

**proc** **sort** data=opioid6; by study_ID drug_number; **run**;

**proc** **sql**;

create table opioid7

as select * from record_total_for_merge

left join opioid6

on record_total_for_merge.identifier=opioid6.identifier;

**quit**;

**proc** **sort** data=opioid7; by study_ID drug_number; **run**;

/* Transpose data from wide to long: drug name, quantity, days supplied, start time*/

**proc** **transpose** data=opioid7 out=wide_drug_name prefix=drug_name;

by study_ID;

ID drug_number;

var din_desc;

**run**;

**proc** **transpose** data=opioid7 out=wide_drug_strength prefix=strength;

by study_ID;

ID drug_number;

var strength;

**run**;

**proc** **transpose** data=opioid7 out=wide_dosage_form prefix=dosage_form;

by study_ID;

ID drug_number;

var dosage_form;

**run**;

**proc** **transpose** data=opioid7 out=wide_drug_quantity prefix=quantity;

by study_ID;

ID drug_number;

var quantity_rev;

**run**;

**proc** **transpose** data=opioid7 out=wide_dayssupl prefix=dayssupl;

by study_ID;

ID drug_number;

var dayssupl;

**run**;

**proc** **transpose** data=opioid7 out=wide_days_to_dt_of_serv_ts prefix=days_to_dt_of_serv_ts;

by study_ID;

ID drug_number;

var days_to_dt_of_serv_ts;

**run**;

**proc** **transpose** data=opioid7 out=wide_start_date prefix=start_date;

by study_ID;

ID drug_number;

var start;

**run**;

**proc** **transpose** data=opioid7 out=wide_end_date prefix=end_date;

by study_ID;

ID drug_number;

var end_date;

**run**;

**data** wide_combined;

merge wide_drug_name wide_drug_quantity wide_dayssupl wide_days_to_dt_of_serv_ts wide_start_date wide_end_date;

by study_ID;

**run**;

**data** wide_combined4_0;

set wide_combined;

array f {**98**} start_date1-start_date98;

array g {**98**} end_date1-end_date98;

array h {**98**} lengths1-lengths98;

array j {**98**} begin1-begin98;

array k {**98**} finish1-finish98;

array l {**98**} L1-L98;

/*** only calculate for those who have used drug within 1 year post transplant.

set others to missing, so we do not include those in either opioid group or non-opioid group ***/

do i=**1** to **98**;

if f(i)>**0** and g(i)<**365** and g(i)>**0** then do; h(i)=g(i) - f(i); j(i)=f(i); k(i)=g(i); end;

else if f(i)<=**0** and f(i) >**.** and g(i)<**365** and g(i)>**0** then do; h(i)=g(i); j(i)=**0**; k(i)=g(i); end;

else if f(i)<=**0** and f(i) >**.** and g(i)>=**365** then do; h(i)=**365**; j(i)=**0**; k(i)=**365**; end;

else if f(i)>**0** and f(i)<**365** and g(i)>=**365** then do; h(i)=**365**-f(i); j(i)=f(i); k(i)=**365**; end;

end;

do i=**1** to **98**;

l(i) = g(i) - f(i);

end;

drop i;

**run**;

**data** wide_combined4_1;

set wide_combined4_0;

total_supply=sum(of dayssupl1-dayssupl98);

earliest_start=min(of start_date:);

latest_end=max(of end_date1-end_date98);

lengths_within_1yr_post=sum(of lengths:);

begin_within_1yr_post=min(of begin:);

end_within_1yr_post=max(of finish:);

L = sum(of L1-L98);

**run**;

/* define opioid user vs non user */

**data** wide_combined4;

set wide_combined4_1;

length user_type $100.;

if lengths_within_1yr_post>=**30** then user_type="Opioid user (>=30 days usage within 1 year post BMT)";

else if lengths_within_1yr_post<**30** and lengths_within_1yr_post>=**0** then user_type="Used opioid >0 and <30 days within 1 year post BMT";

else if lengths_within_1yr_post=**.** and L>**0** then user_type="No usage within 1 year post BMT; but used opioids entirely before BMT or after 1 year post BMT";

else user_type="Non opioid user";

length user_type2 $100.;

if lengths_within_1yr_post>=**60** then user_type2="Opioid user (>=60 days usage within 1 year post BMT)";

else if lengths_within_1yr_post<**60** and lengths_within_1yr_post>=**0** then user_type2="Used opioid >0 and <60 days within 1 year post BMT";

else if lengths_within_1yr_post=**.** and L>**0** then user_type2="No usage within 1 year post BMT; but used opioids entirely before BMT or after 1 year post BMT";

else user_type2="Non opioid user";

length user_type3 $100.;

if lengths_within_1yr_post>=**90** then user_type3="Opioid user (>=90 days usage within 1 year post BMT)";

else if lengths_within_1yr_post<**90** and lengths_within_1yr_post>=**0** then user_type3="Used opioid >0 and <90 days within 1 year post BMT";

else if lengths_within_1yr_post=**.** and L>**0** then user_type3="No usage within 1 year post BMT; but used opioids entirely before BMT or after 1 year post BMT";

else user_type3="Non opioid user";

**run**;

/****************************************************************/

/************** merge datasets *********************************/

/**************************************************************/

**proc** **sort** data=wide_combined4; by study_ID; **run**;

**proc** **sort** data=data.master3 ; by study_ID; **run**;

**proc** **sort** data=record_total_update; by study_ID; **run**;

**data** master_update;

set data.master3;if diagnose in ("Myeloma", "DCN", "GATA2 Deficiency", "MF", "MPN", "Non-Malign", "SAA") then delete;

**run**;

**proc** **sql**;

create table master_opioid_prep

as select * from master_update

left join wide_combined4

on wide_combined4.study_id=master_update.study_id;

**quit**;

**proc** **sql**;

create table master_opioid_prep2

as select * from master_opioid_prep

left join record_total_update

on master_opioid_prep.study_id=record_total_update.study_id;

**quit**;

**data** master_opioid;

set master_opioid_prep2;

length user $100.;

length user_final3 $100.;

if user_type="Opioid user (>=30 days usage within 1 year post BMT)" and earliest_start<**0**

then user_final3="";

else if user_type="Opioid user (>=30 days usage within 1 year post BMT)" and earliest_start>=**0**

then user_final3="Opioid user";

else if user_type="Used opioid >0 and <30 days within 1 year post BMT" and

study_ID in ("xxxxx", "xxxxx","xxxxx", "xxxxx", "xxxxx", "xxxxx", "xxxxx", "xxxxx", "xxxxx",

"xxxxx","xxxxx", "xxxxx", "xxxxx" ) then user_final3="Opioid user";

else if user_type="Used opioid >0 and <30 days within 1 year post BMT" and

study_ID not in ("xxxxx", "xxxxx","xxxxx", "xxxxx", "xxxxx", "xxxxx", "xxxxx", "xxxxx", "xxxxx",

"xxxxx","xxxxx", "xxxxx", "xxxxx" ) then user_final3="Non opioid user";

else if user_type="No usage within 1 year post BMT; but used opioids entirely before BMT or after 1 year post BMT" and earliest_start<**0**

then user_final3="Non opioid user";

else if user_type="No usage within 1 year post BMT; but used opioids entirely before BMT or after 1 year post BMT" and earliest_start>**0**

then user_final3="";

else if user_type="Non opioid user" then user_final3="Non opioid user";

else user_final3="Non opioid user";

if begin_within_1yr_post=**.** then begin_within_1yr_post2=**0**;

else begin_within_1yr_post2=begin_within_1yr_post;

**run**;

**data** master_opioid2;

set master_opioid;

if latest_end<=**0** then grouping="Before";

else if latest_end>**0** then grouping="After";

if therapy_related ne "" then therapy_related_rev="Yes";

else therapy_related_rev="No";

/* aGVHD 3-4*/

length aGVHD34 $100.;

if agvh_grade in ("","N/A","grade1","grade2") then aGVHD34="No aGVHD or Grade 1-2 aGVHD";

else if agvh_grade in ("grade3","grade4") then aGVHD34="Grade 3-4 aGVHD";

if days_to_agvhd_date>**0** and agvh_grade in ("grade3","grade4") then days_to_agvhd2=days_to_agvhd_date;

/* aGVHD 2-4*/

length aGVHD24 $100.;

if agvh_grade in ("","N/A","grade1") then aGVHD24="No aGVHD or Grade 1 aGVHD";

else if agvh_grade in ("grade2","grade3","grade4") then aGVHD24="Grade 2-4 aGVHD";

if days_to_agvhd_date>**0** and agvh_grade in ("grade2", "grade3","grade4") then days_to_agvhd24_2=days_to_agvhd_date;

/* include no cGVHD and mild cGVHD */

length cGVHD_grade_rev $100.;

if cGVH_grade in ("Mild","mild","") then cGVHD_grade_rev="No cGVHD or Mild cGVHD";

else if cGVH_grade in ("Moderate to Severe","Severe","grade2","moderate","moderate to severe","severe") then cGVHD_grade_rev="Moderate to Severe cGVHD";

if days_to_cgvhd_date>**0** and cGVH_grade in ("Moderate to Severe","Severe","grade2","moderate","moderate to severe","severe")

then days_to_cgvhd2=days_to_cgvhd_date;

/* only include mild cGVHD */

length cGVHD_grade_rev2 $100.;

if cGVH_grade in ("Mild","mild") then cGVHD_grade_rev2="Mild cGVHD";

else if cGVH_grade in ("Moderate to Severe","Severe","grade2","moderate","moderate to severe","severe") then cGVHD_grade_rev2="Moderate to Severe cGVHD";

if study_id="xxxxx" then user_final3="Non opioid user";

**run**;

**data** master_opioid3_0;

set master_opioid2;

if user_final3="Opioid user" and earliest_start>**0** then do;

days_to_last_f_u2=days_to_last_f_u-begin_within_1yr_post2;

days_to_relapse_date2=days_to_relapse_date-begin_within_1yr_post2;

days_to_agvhd34_date2=days_to_agvhd2-begin_within_1yr_post2;

days_to_agvhd24_date2=days_to_agvhd24_2-begin_within_1yr_post2;

days_to_cGVHD_mod_sev_date2=days_to_cgvhd2-begin_within_1yr_post2; end;

/* 13 patients qualify as opioid user, they started before BMT or within 1 year post BMT,

within first 1 year post BMT they used opioids, total duration<30 days

HOWEVER THEY DIED ON OPIOIDS. */

else if (user_final3="Opioid user" and earliest_start<=**0**) then do;

days_to_last_f_u2=days_to_last_f_u;

days_to_relapse_date2=days_to_relapse_date;

days_to_agvhd34_date2=days_to_agvhd2;

days_to_agvhd24_date2=days_to_agvhd24_2;

days_to_cGVHD_mod_sev_date2=days_to_cgvhd2; end;

else if user_final3="Non opioid user" then do;

days_to_last_f_u2=days_to_last_f_u-**95**;

days_to_relapse_date2=days_to_relapse_date-**95**;

days_to_agvhd34_date2=days_to_agvhd2-**95**;

days_to_agvhd24_date2=days_to_agvhd24_2-**95**;

days_to_cGVHD_mod_sev_date2=days_to_cgvhd2-**95**; end;

**run**;

**data** master_opioid3_0_check;

set master_opioid3_0;

keep study_id user_final3 begin_within_1yr_post2 earliest_start days_to_relapse_date days_to_relapse_date2 begin_within_1yr_post2 begin_within_1yr_post;

where user_final3="Opioid user" and days_to_relapse_date2<**0** and days_to_relapse_date2 ne **.**;

**run**;

**data** master_opioid3_1;

set master_opioid3_0;

days_to_last_f_u3=days_to_last_f_u2 ;

/* some patients had relapse or aGVHD or cGVHD before they BMT... do not count these events */

if days_to_relapse_date2<**0** and days_to_relapse_date2 ne **.**

then do; relapse3=**.**; days_to_relapse_date3=**.**; end; else do; relapse3=relapse; days_to_relapse_date3=days_to_relapse_date2; end;

length aGVHD34_indicator3 $100.;

length acvhd_organ_involvement3 $100.;

if days_to_agvhd34_date2<**0** and days_to_agvhd34_date2 ne **.**

then do; aGVHD34_indicator3=""; acvhd_organ_involvement3=""; days_to_agvhd34_date3=**.**; end;

else do; aGVHD34_indicator3=aGVHD34; acvhd_organ_involvement3=acvhd_organ_involvement; days_to_agvhd34_date3=days_to_agvhd34_date2; end;

length aGVHD24_indicator3 $100.;

length acvhd_organ_involvement3 $100.;

if days_to_agvhd24_date2<**0** and days_to_agvhd24_date2 ne **.**

then do; aGVHD24_indicator3=""; acvhd_organ_involvement3=""; days_to_agvhd24_date3=**.**; end;

else do; aGVHD24_indicator3=aGVHD24; acvhd_organ_involvement3=acvhd_organ_involvement; days_to_agvhd24_date3=days_to_agvhd24_date2; end;

length cGVHD_grade_rev_3 $100.;

length cGVHD_grade_rev2_3 $100.;

length cGVHD_organ3 $100.;

if days_to_cGVHD_mod_sev_date2<**0** and days_to_cGVHD_mod_sev_date2 ne **.**

then do; days_to_cGVHD_mod_sev_date3=**.**; cGVHD_organ3="";

cGVHD_grade_rev_3=""; cGVHD_grade_rev2_3=""; end;

else do; days_to_cGVHD_mod_sev_date3=days_to_cGVHD_mod_sev_date2; cGVHD_organ3=cGVHD_organ;

cGVHD_grade_rev_3=cGVHD_grade_rev; cGVHD_grade_rev2_3=cGVHD_grade_rev2; end;

/*** non users: some were stopped to follow up before day 95, remove these patients ***/

if days_to_last_f_u3<**0** then delete;

**run**;

/* time to relapse */

**proc** **means** data=master_opioid3_1 median min max maxdec=**5**; class user_final3; var days_to_relapse_date; where relapse=**1** and days_to_relapse_date3 ne **.**; **run**;

**proc** **npar1way** data=master_opioid3_1 wilcoxon; class user_final3;var days_to_relapse_date; where relapse=**1** and days_to_relapse_date3 ne **.**; **run**;

**data** master_opioid3_1_check;

set master_opioid3_1;

keep study_id user_final3 days_to_agvhd_date agvh_grade days_to_agvhd34_date days_to_agvhd34_date2 days_to_agvhd34_date3 aGVHD34_indicator3 aGVHD34 days_to_last_f_u2;

**run**;

**proc** **means** data=master_opioid3_1 min mean median max; class user_final3; var days_to_last_f_u3 days_to_relapse_date3 days_to_agvhd34_date3 days_to_cGVHD_mod_sev_date3; **run**;

**proc** **freq** data=master_opioid3_1; tables dead relapse3 aGVHD34_indicator3 aGVHD24_indicator3 cGVHD_grade_rev_3; **run**;

**data** master_opioid3;

set master_opioid3_1;

/**** Relapse ****/

if days_to_relapse_date3 ne **.** then do; relapse_ind=**1**; relapse_days=days_to_relapse_date3; end;

else if days_to_relapse_date3=**.** and dead=**1** then do; relapse_ind=**2**; relapse_days=days_to_last_f_u3; end;

else if days_to_relapse_date3=**.** and dead=**0** then do; relapse_ind=**0**; relapse_days=days_to_last_f_u3; end;

/*** Relapse Free Survival/Treatment Related Mortality ***/

if dead=**1** and relapse3 in (**0**,**.**) then do; RFS=**1**; RFS_days=days_to_last_f_u3;end;

else if dead=**1** and relapse3=**1** then do;RFS=**1**; RFS_days=days_to_relapse_date3;end;

else if dead=**0** and relapse3=**1** then do;RFS=**1**; RFS_days=days_to_relapse_date3;end;

else if dead=**0** and relapse3 in (**0**,**.**) then do;RFS=**0**; RFS_days=days_to_last_f_u3;end;

/*** aGVHD 3-4 ***/

if aGVHD34_indicator3="Grade 3-4 aGVHD" then do; aGVHD=**1**; aGVHD_days=days_to_agvhd34_date3;end;

else if dead=**1** and aGVHD34_indicator3 ne "Grade 3-4 aGVHD" then do; aGVHD=**2**; aGVHD_days=days_to_last_f_u3;end;

else if dead=**0** and aGVHD34_indicator3 ne "Grade 3-4 aGVHD" then do; aGVHD=**0**; aGVHD_days=days_to_last_f_u3;end;

/*** aGVHD 2-4 ***/

if aGVHD24_indicator3="Grade 2-4 aGVHD" then do; aGVHD24=**1**; aGVHD24_days=days_to_agvhd24_date3;end;

else if dead=**1** and aGVHD24_indicator3 ne "Grade 2-4 aGVHD" then do; aGVHD24=**2**; aGVHD24_days=days_to_last_f_u3;end;

else if dead=**0** and aGVHD24_indicator3 ne "Grade 2-4 aGVHD" then do; aGVHD24=**0**; aGVHD24_days=days_to_last_f_u3;end;

if aGVHD34_indicator3="Grade 3-4 aGVHD" then time_aGVHD=days_to_agvhd34_date3;

if aGVHD24_indicator3="Grade 2-4 aGVHD" then time_aGVHD24=days_to_agvhd24_date3;

/*** cGVHD ***/

if cGVHD_grade_rev_3="Moderate to Severe cGVHD" then do; cGVHD=**1**; cGVHD_days=days_to_cGVHD_mod_sev_date3;end;

else if dead=**1** and cGVHD_grade_rev_3 ne "Moderate to Severe cGVHD" then do; cGVHD=**2**; cGVHD_days=days_to_last_f_u3;end;

else if dead=**0** and cGVHD_grade_rev_3 ne "Moderate to Severe cGVHD" then do; cGVHD=**0**; cGVHD_days=days_to_last_f_u3;end;

if cGVHD_grade_rev="Moderate to Severe cGVHD" then time_cGVHD=days_to_cGVHD_mod_sev_date3;

/*** non-relapse mortality ***/

if dead=**1** and relapse3 in (**0**,**.**) then do; NRM=**1**; NRM_days=days_to_last_f_u3;end;

else if dead=**1** and relapse3 = **1** then do; NRM=**2**; NRM_days=days_to_relapse_date3;end;

else if dead=**0** and relapse3 = **1** then do; NRM=**2**; NRM_days=days_to_relapse_date3;end;

else if dead=**0** and relapse3 in (**0**,**.**) then do; NRM=**0**; NRM_days=days_to_last_f_u3;end;

/** GVHD free, relapse free survival (GRFS) ***/

if dead=**1** or relapse3=**1** or aGVHD34_indicator3="Grade 3-4 aGVHD" or cGVHD_grade_rev_3="Moderate to Severe cGVHD"

then do; GRFS=**1**;

GRFS_days=min(days_to_last_f_u3, days_to_relapse_date3, days_to_agvhd34_date3, days_to_cGVHD_mod_sev_date3); end;

else do; GRFS=**0**; GRFS_days=days_to_last_f_u3; end;

keep IndexYear study_id age_group therapy_related_rev diagnose donor gender stage source RIC_MAC aGVHD34_indicator3 days_to_agvhd34_date3 acvhd_organ_involvement3

cgvhd_organ3 cGVHD_grade_rev_3 cGVHD_grade_rev2_3 days_to_cGVHD_mod_sev_date3 dead grouping user total_days time_aGVHD time_aGVHD24 time_cGVHD

cytogenetics molecular relapse3 days_to_relapse_date3 donor_type hla_match_0001 match_level

kps_prior_sct hct_ci dri_0_low___1_intermediate_2_hi Length_of_follow_up earliest_start latest_end Length_of_follow_up days_to_last_f_u3

cause_of_death cause_of_death__primary_ cause_of_death__secondary_ /*total_days_above_1month intense_user*/ user

days_to_last_f_u relapse days_to_relapse_date gvh_prophylaxis

relapse_ind RFS agvh_grade aGVHD aGVHD24 cGVHD NRM relapse_days RFS_days aGVHD_days aGVHD24_days cGVHD_days NRM_days user_final3 GRFS GRFS_days days_to_discon_of_immuno;

**run**;

**proc** **freq** data=master_opioid3; tables gvh_prophylaxis*donor; **run**;

**proc** **freq** data=master_opioid3; tables agvh_grade; **run**;

/*****************************************************************************************************/

/* collapse the categories for cytogenetics, molecular and staging */

/*****************************************************************************************************/

/* clean up cytogenetics categories */

**data** master_opioid4;

set master_opioid3;

if cytogenetics in ("inv 16, c-kit mutation exon 8 t(8:21)"," inv 16,c-kit mutation exon 8 t(8:21)", "45 x -Y t(8;21)", "45,X,-X,t(8;21)(q22;q22)[20]",

"45,X,-Y,t(8;21)(q22;q22)[6]/46,XY,t(8;21)(q22;q22", "46 XX, t (8;21)(q22;q22)" , "46,XX,ins(8;21)(q22;q22.1q22.3)[20]",

"46,XX,inv(16)(p13.1q22)[20]", "46,XX,t(8;21)(q22;q22)", "46,XX,t(8;21)(q22;q22)[3]/46,XX[1]", "46,XY,inv(16)(p13.1q22)",

"46,XY,inv(16)(p13.1q22)[10]", "46,XY,t(15;17)(q24;q21)[10]", "46XX, t(8;21)", "47,XX,+8,t(8;21)(q22;q22)[", "47,XY,+8,t(8;21)(q22;q22)[10]",

"inv 16, +22", "t (8;21)") then cyto_code=**0**;

else if cytogenetics in ("inv 16, c-kit mutation exon 8 t(8:21)", "11q del", "11q del pos, tri 12 neg, monosomy 13 neg, 13qdel neg, 17 p del neg",

"13q, 17p deletion", "17P", "17p", "17p 11q", "17p and 13q", "17p del, + and ++12, +and ++13, +and ++11", "17p deletion, 13q deletion",

"17p inconclusive, 13q +, 11q -, trisomy 12 neg",

"34<1n>,X,+X,+1,+5,+6,+8,+10,+11,+12,+14,+18,+21[4]/62,slx2,-6,-10,-11,-14[cp14]/46,XX[6]",

"42-45 XY, -3, -5, -7, -13, -18, -20, -21",

"43,XY,der(5;17)(p10;q10),-7,add(12)(q21-23),del(13)(q12q22),-15,-16,del(20)(q11.2q13.3),+mar1,+mar2[cp9]",

"43-44, XY, -5, add (5) (q11.2), add (6) (q21), -7, -8, -9, .-12, -18, -21, +2-4 (ep19) -46 XY.",

"44,X,X,add(1)(p13),5,der(17)t(?9;17)(p13;p11.2),18,der(20)t(1;20)(p22;q11.2),+add(21)(q22);4idem,t(2;3)(p21;p(13);46,XX",

"44,XX,-7,der(13;14)",

"44,XX,add(3)(q21),-4,-5,-6,?del(17)(p11.2),+mar1[4]/44,XX,add(3)(q21),-4,-5,-6,-17,+mar1,+mar2[4]/46,XX[8], pos for deletion 17p",

"44,XY, Del(5)(q13),add(6)(q27), -18, 20, dlo(22,?)(q13, ?),1-2min(cp12]",

"45 X,-Y, add(2)(p12), t(8;21)(q22;q22)", "45,X,-X,t(8;21)(q22;q22),del(9)(q13q22)[10]",

"45,X,-Y,t(6;9)(p22;q34),-13,add(13)(p11.2),-14,-22,+mar1,+mar2,+mar3[14]/46,XY[6]", "45,XX,-4,-7,+mar[9]/45,sl,del(6)(q21q23)[2] (incomplete complex)",

"45,XX,der(6)add(6)(p21)add(6)(q25),add(7)(q11.2),-12[11]/46,XX[4]", "45,XY,-3,del(5)(q22q31),i(11)(q10)[11]/44,idem,dic(12;14)(p11.2;p12)[4]/46,XY[5]",

"45,XY,-5,del(7)(q22)[4]/44,sl,-21[4]/45,sl,t(15;21)(q15;p13)[4]", "45,XY,-7[12]/46,XY[8]",

"45,XY,-7[14]/46,XY,t(9;22)(q34;q11.2)[6]/46,XY[3]", "45,XY,-7[1]/46,XY[19].",

"45,XY,-7[5]/46,idem,+mar[5]/46,XY,del(7)(q22)[3]/46,XY7q del", "45,XY,-7[8]/45,sl,t(6;7)(p25;q11.2)[10]",

"45,XY,add(3)(p21),-5,del(9)(q13q22)[9]/46,XY[7]", "45,XY,del(5)(q22q32),-13[18", "45,XY,del(5)(q22q35),t(12;14)(q24.1q32),-17,add(17)(p13)[9]/46,XY[1]",

"45,XY,der(2;6)(p10;p10),del(5)(q22q31),der(6)t(2;6)(q21;q27),-7,del(16)(q12-13),-20,add(20)(p13),add(21)(p11.2),+22,+mar[12]/44,idem,-18[5]",

"45,XY,inv(3)(q21q26.2),-7[20]/46,XY[2]", "45XX, -7,del 17(q22-q24)", "46 XX with 11q23[5]", "46 XY, del 17p",

"46, XY, del (5), (q13q33), del (5) (q?13q?33), del (7) (q11,2), +0, add (11) (p15), -15, add (15) (q15) (cp11)/46, XY(1)",

"46, XY,t(10;11)p(13:q21)[10]/46,XY(9)", "46,X,der(X)t(X;1)(p22.1;q12)",

"46,X,t(Y;1)(q11.2;q25),del(2)(p11.2p13),add(4)(q12),t(6;14)(p22;q32),i(9)(q10),add(13)(q22),-16,add(17)(p13), +mar[7]/46,idem,t(X;19)(p10;p10),+2,-del(2)(p11.2p13),-7,+r[5]",

"46,XX,+1,der(1;7)(q10;p10)[9]/47,idem,+8", "46,XX,+1,der(7)t(1;7)(q10;p10)",

"46,XX,add(2)(p11.2),add(7)(q11.2),t(7;9)(q36;q32),-20,i(21)(q10),del(22)(q13),+mar[10]",

"46,XX,del(5) (q13q33), del(18), (q21) [8]/46,XX t(3;10) (q26;q21), t(4;12) (q12;p13)[2]", "46,XX,del(5)(q13q31)[6]",

"46,XX,del(5)(q13q33)[3]", "46,XX,del(5)(q15q31)[6]/46,XX[5]", "46,XX,del(5)(q15q33)[10]/sl,t(12;20)(q13;q13.1)[9]",

"46,XX,del(5)(q15q33)[16]/47,XX,+8[2]/46,XX[2]", "46,XX,del(5)(q15q33)[1]/48,idem,add(3)(p13),+del(5)x2,add(16)(p13.1),add(19)(p13),-21[4]/46,XX[6]",

"46,XX,del(7)(q11.2q22)[4]/46,XX,del(20)(q11.2-13.1)[4]/46.XX[12]", "46,XX,del(7)(q22)",

"46,XX,der(9)t(9;22)(q34;q11.2),idic der(22)t(9;22)(q34;q11.2)[15]/47,XX,t(9;22)(q34;q11.2),+der(22)t(9;22)[9]",

"46,XX,t(11;19)(q23;p13.1)[13]", "46,XX,t(11;19)(q23;p13.1)[20]",

"46,XX,t(9;22)(q34;q11.2", "46,XX,t(9;22)(q34;q11.2)", "46,XX,t(9;22)(q34;q11.2)[", "46,XX,t(9;22)(q34;q11.2)[13]",

"46,XX,t(9;22)(q34;q11.2)[14]/50,idem,+10,+17,+21,+der(22)[6]", "46,XX,t(9;22)(q34;q11.2)[18]/46,XX[2]", "46,XX,t(9;22)(q34;q11.2)[19]/46,XX[1]",

"46,XX,t(9;22)(q34;q11.2)[22]", "46,XX,t(9;22)(q34;q11.2)[9]", "46,XY,del(2)(p11.2),+add(2)(q11.2),del(5)(q15q33),-10,-15,+mar[4]/46,XY[",

"46,XY,del(3)(p21),+del(3)(q21),+del(6)(q21),del(7)(q22q32),-11,-14,-17,+mar[9]","46,XY,del(5)(q11.2q35),-17,+r[18]/46,XY[2]",

"46,XY,del(5)(q13q33)[10]/46,XY[10]","46,XY,del(5)(q15q33)[15]/46,XY[5]", "46,XY,del(5)(q22q35)[10]", "46,XY,del(7)(q22),del(20)(q11.2)",

"46,XY,del(7)(q22),inv(16)(p13q22)[10]","46,XY,del(7)(q22)[9]/46,XY[2]","46,XY,del(7)(q22q22)[3]/47,idem,+13[10]","46,XY,der(1;7)(q31;q22)[3]/46,XY[1]",

"46,XY,der(7;10)(p10;q10),+10[8]/46,XY,der(7)r(7;?10)(p22p10;q10q26)[4]/47,XY,+8[10]",

"46,XY,dup(2)(q11.2q24),add(9)(q34)[19]/46,XY,dup(2)(q11.2q24),add(8)(q24)[4]/46,XY,dup(2)(q11.2q24), add(4)(q35)[3]/46,XY,dup(2)(q11.2q24),add(12)(q24.3)[2]/46,XY,dup(2)(q11.2q24),add(15)(q26.3)[2]/46,XY[7]",

"46,XY,t(2;11)(p21;q23)[1]/46,idem,del(5)(q15q33)[19]", "46,XY,t(9;22)(q34;q11.2", "46,XY,t(9;22)(q34;q11.2)",

"46,XY,t(9;22)(q34;q11.2) (double Ph' chromosome)", "46,XY,t(9;22)(q34;q11.2)[", "46,XY,t(9;22)(q34;q11.2)[13]",

"46,XY,t(9;22)(q34;q11.2)[14]/55,XY,+X,+Y,+4,+6,t(9;22)(q34;q11.2),+14,+15,+18,+21,+der(22)t(9;22)[6]",

"46,XY,t(9;22)(q34;q11.2)[1]/46,idem,t(2;3)(q23;q29)[9]", "46,XY,t(9;22)(q34;q11.2)[21]", "46,XY[10].ish ?del(11)(q23)(MLL+)[2]",

"46,XY[20], ADD (7) (P13), t(9;14) (q.34;q.22),? DEL (11) (q.21, q.23),", "46XX, (del(5)(q15q31), del(9)(q13q22) and 48XX, idem +6, +8",

"46XX, 5q-, t(3:14)", "46XX, t(9;22)(q34;q11.2)", "46XY, -5, del(7)(q22), add(11)(q25), -14, -18, +R1, +R2, +R3, MAR (CP14)",

"46XY, add(5)(q22) (del of material distal to 5q22)", "46XY, t(9;22), -3, -7, add 8, add9", "47 XX, del 7q, +8",

"47, XY, +9, del (12), (p112.p13) [14]/48, sl, add(5) (q?31), +6 [4]/46, XY [4]",

"47,XX,+8,ins(9;11)(p22;q13q23)[12].ish ins(9;11)(p22;q13q23)(5'MLL+;3'MLL+)/46,XX[2], Trisomy 8","47,XX,+9,der(21)t(1;21)(q21;p10)",

"47,XY, +der(9)t(1;9)(q12;q12)[11]/47,XY, +9[9].", "47,XY,+19[3]/48,idem,+8[6]/49,idem,+8,+13[6]", "47,XY,+8",

"47,XY,add(1)(p36),t(4;11)(q21;q23),+7[9]/46,XY[1]", "47XX, 13q del, 17p del, +12", "47XY, t(1;14)(p32;q11.2), inv(8)(p23;q24), +21",

"47XY, t(3;13;5)(p21,q14,p13), t(8.14)(q24.1;q32), del(9)(p13p22), t(14;18)(q32;q21)","47XY, t(9;22)(q34;q11.2), +8",

"48,XX,+8,+21[8]/49,idem,+19[6]/50,idem,+9,+19[5]/46,XX[4]", "48,XX,+8,+9[13]/48,XX,der(1)t(1;9)(q21;q13),+8,+der(9)t(1;9)(q21;q21)x2[7]",

"48,XX,inv(16)(p13.1q22),+22,+mar[9]/46,XX[1]", "48,XY,del(5)(q13q33),+8,del(8)(q22),-20,+2mar[8]/46,XY[3]",

"48,XY,der(2)t(2;2)(q33;p13),der(2)t(2;15)(p13;q11.2),+8,+13[19]/46,XY[1]",

"48,XY,t(6;22)(p21;q13),?t(16;16)(p13.1;q22),-18,+der(22)t(6;22)(p21;q13),+mar1,+mar2[4]/46,XY[3]",

"48,X,-X,del(2)(p23),t(2;12;6)(q35;q13;q21),del(3)(q12q21),-4,del(5)(q13),-6,add(7)(q36),add(8)(p11.2),+add(8)(p11.2),+i(8)(q10),-17,-18,+19,+21,+add(22)(q13),+2ar[cp10]",

"49,XY,+X,+8,+10[11]",

"49,XY,-7,+8,+11,+13,add(17)(p11.2),+22[4], 45,XY,-7,t(12;21)(q22;q22),add(17)(p11.2), 46 XY, Complext including monosomy 7",

"49XX, +5, +8, ider(9)(q10), t(9;22)(q34;q11.2),der(22) t(9;22), +der(22) t(9;22)",

"51,XY,inv(3)(q2?3q26),+8,t(9;22)(q34;q11.2),+19,+20,+21,+der(22)t(9;22)[15]/46,XY[1]",

"52,XY,+8,+8,+i(9)(q10),t(11;14)(q23;q32),+der(11)t(11;14),+16,+19[3]/52,idem,dup(17)(q21q25)[6]/46,XY[3]",

"52,XY,+X,+8,+9,+11,+12,+13", "5p del", "5q del - done outside", "5q del, 7q del (FISH)",

"6,XY,t(16;16)(p13.1;q22),add(17)(q11.2)[12]/46,idem,del(2)(q31),add(7)(q22)[5]/46,XY[3]",

"80<4n>,XXY,-Y,add(1)(p36),-2,add(2)(q31)x1, -3,add(3)(q12)x2,-6,-7,der(7)t(7;8)(p15;q13),-8,-8, -9,-9,add(12)(p11.2)x2,-13,-13,-15,-15,-16,-16,-17,-17, -18,+3ar[cp9]",

"90,<4n>,XXX,-X,-2,+6,+6,-7,t(11;17)(q23;q21),der(11)t(11;17),-17[cp8]/46,XX[2]",

"Complex", "Complex Karyotype incl. Monosomy 7", "Complex cytogenetics", "Complex cytogenetics - done outsideb 46XX, del(3)(q27), t(6;21)(q25;q22), -8,+add (11)(q13), complex",

"Monosomy 7", "Monosomy 7, del 13q, missing Y", "Phi", "Phi +", "complex", "complex cytogenetics", "complex cytogenetics at myeloid blast crisis, 46 XY after R, 2.5 log reduction",

"complex cytogenetics incl -7, 17p rearr, 3q rearr", "complex including monosomal", "complex karyotype including 5,q deletion, 7q deletion, and 17p deletion.",

"complex karyotype, 2 clones, (incl -5,-7,+8,-10, -12, -14, -16, +21)", "complex karyotype, monosomal changes", "complex, t(9;22)(q34;q11.2)",

"del (5) (q31;q35) done outside","del 7, del 9", "del 7q", "del 7q, t16;16)", "del 7q34",

"der(9)t(1;9),del(10)(p11.2)[10]/52,XY,t(1;3)(p22;q27),+der(1)t(1;3),+6,+8,+9,del(10)(p11.2),+13,+19,+22[cp2]/46,XY[7]",

"loss: 1q, 8p, 11q, gain: 8q, 17q", "t (11;19)", "t (4;11) t (7;10) FISH MLL Pos", "t (9;11)", "t (9;22) - done outside",

"t(9;11)", "t(9;22)","translocation(5:7); additional 17, -20",

"41,XY,der(4;5)(p10;p10),+del(7)(q11.2),der(?7;17)(p10;q10),?dic(13;?21)(p13;q22),-14,-15, der(16)t(16;17)(p11.2;q21)t(14;16)(q11.2;q13),-17,-18,-20,+2r,+1ar",

"35,XY,-3,del(3)(q21),-5,-6,del(6)(q21),-7,add(9)(q34),der(9)t(9;13)(q32;q14),-14,der(14;22)(q10),-20,-21,add(21)(p13),+r,+1-3mar , Complext including monosomy 7",

"42,XY,der(1)t(1;11)(p32;q13),-3,add(4)(q12),-5,-7,-9,t(10;16)(p13;q22),der(11)t(11;?)(q13;?),.-12,add(15)(q24),-18,+3ar[cp13]/46,XY[2]",

"42,XY,del(2)(q31q33),-3,-4,-5,del(5)(q13q31),-7,inv(12)(p12p13),add(17)(p11.2),-18[cp10]",

"44,XX,-3,-5,t(6;18)(q27;q21.1),-7,+21,+22,+mar[cp10]",

"45,Y,add(X)(p11.2),+Y,+1,add(5)(q22),+8,+10,+11, -18,+19,-20,del(20)(q13.1)x2,+21,+mar(cp10), del5q COMPLEX",

"46,XY,del(3)(p21),del(5)(q13q35),add(6)(p22),-12,+21,idic(21)(q22.3),idic(21)(q22.3),+mar[cp6]",

"46,XY,t(1;3)(p22;q27),del(10)(p11.2),+mar[cp3]/46,XY,der(1)t(1;3)(p22;q27),der(3)t(1;3)t(1;9)(p36.3;q22),",

"57,XY,+del(1)(q31),del(5)(q31),der(7)t(1;7)(q31;q11.2),+8,+8,+9,+11,+14,+18,+19,+21,+22,+22[3]/57,idem,-11,+i(11)(q10)[3]/46,XY[7]",

"Complex del 6q, transl Chr 10,14,17,18", "+8, +11, +x", "MONOSOMY 5 AND 7", "complex karyotype including 5q deletion, 7q deletion, and 17p deletion.",

"45,X,add(X)(q13),del(5)(q15q33),add(7)(p13),-9,dic(10;21)(q11.2;q22),add(11)(q13),del(12)(q24.1),+mar[4]dem,del(3)(q13),add(6)(p23)[4]/45,idem,add(2)(q23),add(6)(p23),del(7)(q22)[2]")

then cyto_code=**2**;

else if cytogenetics in ("-Y 3/20", "11qdel", "13 del", "13q del", "34<1n>,X,+X,+1,+5,+6,+8,+10,+11,+12,+14,+18,+21[4]/62,slx2,-6,-10,-11,-14[cp14]/46,XX[6]",

"44-47 X,-X, mar(cp3)", "45 X, -Y", "45, XX, -7[16]", "45,X,-X[5]/46,XX[15]", "45,X,-Y[13]/46,XY[11]",

"45,X,-Y[4]/46,XY[16]", "45,XX,dic(7;12)(p13;p11,2)[19]", "45,XY,t(1;3)(p36.3;q21),-7[10]/46,XY[1]", "46 XX", "46 XX [12]", "46 XX [20]",

"46 XX [20] at diagnosis; suboptimat 46 XX[6] at relpase", "46 XX [21]", "46 XX t(12;22) inv 6", "46 XX[21]", "46 XX[22]", "46 XY",

"46 XY [20]", "46 XY [21]", "46 XY[11] inconclusive", "46, XX", "46, XX [20]", "46, XX[14]", "46, XY [20]", "46, XY [20], Trisomy 8",

"46, XY [8] inconclusive", "46,XX", "46,XX [20]", "46,XX,?del(1)(p36.1),add(7)(q32),t(8;21)(q22;q22)[16]", "46,XX,?del(7)(p15),t(9;22)(q34;q11.2)"

"46,XX,add(10)(p12),?add(11)(p12-14)[11]/46,XX[2]", "46,XX,add(18)(q23)[7]/46,XX,t(6;11)(q27;q23)[5]/46,XX[15]",

"46,XX,add(4)(q35),add(15)(q26)[20]", "46,XX,add(7)(q32)[6]/46,XX[9]", "46,XX,del(20)(q11.2)[5]/46,XX[2]",

"46,XX,del(20)(q11.2-13.1q11.2-13.1)[5]/46,XX[13]", "46,XX,del(8)(q11.2q13.2),t(10;11)(p13;q14)[10]", "46,XX,del(9)(q21)[20]",

"46,XX,dup(2)(q23q14.2),t(8;21)(q22;q22)[14]", "46,XX,dup(8)(q11.2q24.3)", "46,XX,i(9)(q10)[20]", "46,XX,idic(7)(q11.1)[10]",

"46,XX,t(1;16)(q21;112-13h)[3]/46,XX[19]", "46,XX,t(3;12)(p21;q24.1),t(12;13)(p12;q12)", "46,XX,t(3;5)(q23;q33)",

"46,XX,t(6;9)(p23;q34)", "46,XX,t(9;11)(p21;q23)[20]", "46,XX,t(9;11)(p22;q23)[8]/46,XX[6]", "46,XX,t(9;11)(q34;q23)[11]/46,XX[1]",

"46,XX[18]", "46,XX[20]", "46,XX[21]", "46,XX[21];suboptimal 46 XX[12] at relapse","46,XX[24]","46,XY","46,XY trisomy 11","46,XY,+1,der(1;7)(q10;p10",

"46,XY,+1,der(1;7)(q10;p10)","46,XY,add(10)(p13),del(11)(q23q23)[12]","46,XY,add(13)(q12)[8].ish add(13)(D13S319-,D13S25-,LAMP1+++)",

"46,XY,del(20)(q11.2)","46,XY,del(20)(q11.2-13.1q13.3)[15]/46,XY[5]","46,XY,del(20)(q11.2q13.1)[14]/46,XY[6]","46,XY,del(3)(q12q25)[9]",

"46,XY,del(9)(q13q22", "46,XY,i(14)(q10)[11]", "46,XY,t(10;11)(q11.2;q23)[13]/46,XY[2]", "46,XY,t(11;11)(p15;q23)",

"46,XY,t(11;19)(q23;p13.1)[20]/46,XY[1]", "46,XY,t(6;11)(q27;q23)[19]/46,XY[1]", "46,XY,t(7;11)(p15;p15)[10]/46,XY[11]","46,XY,t(9;11)(p22;q23)",

"46,XY,t(9;11)(p22;q23)[9]/46,XY[1]", "46,XY[10]", "46,XY[20]", "46,XY[21]", "46,XY[23]", "46,XY[24]", "46X, -Y, +12?", "46XX", "46XX Inconclusive",

"46XX, +1, der(1;7)(q10;p10), 47XX, +9 (Prev +8 in Ph neg cells", "46XX, Inconclusive", "46XX, Inv 3(constitutional)", "46XX, t(2;12)",

"46XX, t(X;2)", "46XX, unsuccessful", "46XY", "46XY, Diploid karyotype", "46XY, del(20)(q11.2)","46XY, t(11;19)(q23;p13.1)", "46XY, t(6;14(q13;q32).",

"46XY,Chr 22 abnormality", "46XY??","46XYinconclusive", "47 XX, +8", "47 XX,+8", "47 XY, +8", "47 XY, +X", "47,XX +8[4]/46,XX[18]",

"47,XX,+11[8]/46,XX[4]", "47,XX,+4[6]/46,XX[2]", "47,XX,+8", "47,XX,+8,+16[cp3]/46,XX[2]", "47,XX,+8[13]/?47,XX,+11[2", "47,XX,+8[18]/46,XX[2]",

"47,XX,+8[2]/46,XX[18]", "47,XX,+8[2]/46,XX[19]", "47,XX,+8[6]/46,XX[14]", "47,XX,+8[9]/47,XX+15[2]/46,XX[9]", "47,XY,+11[19]/46,XY[1]",

"47,XY,+4[21]/46,XY[1]","47,XY,+8,-13,+mar", "47,XY,+8[5]", "47,XY,+X,t(9;11)(p22;q23", "47XX, +20, t(3,9,11)", "47XX, +21", "47XX, +8, (del)11q",

"47XX, +9, del (20q)", "47XY, +21", "47XY,+12,t(11;14)", "47XY,+add(9)(q13)", "48,XY,+8,+21[15]/46,XY[5]",

"48,XY,add(7)(p22),+8,+8[10] Tetrasomy 8 and 7pdel", "48,XY,t(6;11)(q27;q23),+21,+21", "50XY, +4, +21, +21, der4",

"6,XY,add(2)(q23),add(9)(p13),add(10)(q24)[8]/46,XY[12]", "Failed cytogenetics, By FISH Del(5q) / -5 neg, Del(7q) / -7 neg, KMT2A 11q23 Rearrangement neg",

"Normal karyotype", "Normal karyotype - done outside", "Trisomy 11, 19", "Trisomy 21, Trisomy 14", "Trisomy 21, Trisomy 22", "Trisomy 8",

"Trisomy 8 and 13", "del 9q", "der(7:12)", "normal karyotype - done outside", "pos (del)11q, pos trisomy 12", "suboptimal 46 XX",

"suboptimal 46 XX[12]","suboptimal 46 XX[13]", "suboptimal 46 XX[15]", "suboptimal 46 XY[13]", "suboptimal 46 XY[14]", "suboptimal 46 XY[16]",

"suboptimal 46 XY[5]", "suboptimal 46 XY[8] FISH NEG for 5/7/11q23", "t (1 ; 15) i(1) +1", "t (1;4) inv 9 at relapse",

"t(5;18) done outside", "t(6;17)(p21)(13)", "t(8:14)", "t(9:22)q34, 1:q11.2);BCR-ABL1", "trisomy 21,(del)12p", "trisomy 8",

"trisomy 8, 20;21 rearrangement, del(20q)", "trisomy 9, trisomy 21",

"Inconclusive", "inconclusive","Inconclusive cytogenetics; FISH 5q del positive", "NA", "NA - Intital BM done outside", "No metaphases","Unsuccesfull","Unsuccessful",

"unsuccessful", "cytogenetics unsuccessful", "inconclusive cytogenetics at diagnosis; suboptimel 46 XY[12] at relapse",

"no monosomies by FISH","not available","unsuccessful - from peri blood","","46,XX,t(6;9)(p23;q34)[")

then cyto_code=**1**;

if study_ID="xxxxx" then cyto_code=**2**;

if study_ID="xxxxx" then cyto_code=**2**;

if study_ID="xxxxx" then cyto_code=**2**;

if study_ID="xxxxx" then cyto_code=**2**;

if study_ID="xxxxx" then cyto_code=**2**;

if study_ID="xxxxx" then cyto_code=**2**;

if study_ID="xxxxx" then cyto_code=**0**;

if study_ID="xxxxx" then cyto_code=**1**;

if study_ID="xxxxx" then cyto_code=**1**;

if study_ID="xxxxx" then cyto_code=**2**;

if study_ID="xxxxx" then cyto_code=**1**;

if study_ID="xxxxx" then cyto_code=**2**;

if study_ID="xxxxx" then cyto_code=**2**;

if study_ID="xxxxx" then cyto_code=**2**;

if study_ID="xxxxx" then cyto_code=**2**;

if study_ID="xxxxx" then cyto_code=**2**;

if study_ID="xxxxx" then cyto_code=**2**;

if study_ID="xxxxx" then cyto_code=**2**;

if study_ID="xxxxx" then cyto_code=**2**;

if study_ID="xxxxx" then cyto_code=**2**;

if study_ID="xxxxx" then cyto_code=**2**;

if study_ID="xxxxx" then cyto_code=**1**;

if study_ID="xxxxx" then cyto_code=**2**;

**run**;

**data** master_opioid5;

set master_opioid4;

/*** clean up stage variable ***/

length stage_rev $100.;

if stage in ("CR1") then stage_rev="CR1";

else if stage in ("CR2") then stage_rev="CR2";

else if stage in ("CR3") then stage_rev="CR3";

else if stage in ("Chemo responsive", "Chemosensitive", "Chemosensitive disease", "PR", "Partial response", "Stable disease", "VGPR", "good response")

then stage_rev="Partial response";

**run**;

**data** master_opioid6;

set master_opioid5;

length molecular_rev $100.;

/*** clean up molecular variable ***/

if molecular in ("KIT positive, RUNX1-RUNX1T1 + c-kit", "C-kit mutation, increasing titres", "CBFB-MYH11, c-kit",

"FLT3-ITD neg, FLT3-TKD neg, NPM1 neg, c-kit pos, RAD21,CUX1 mutations", "NPM1 neg, FLT3-ITD neg, RUNX1 pos, c-KIT pos, RUNX1-RUNXTI",

"RNX1-RUNX1 and C-KIT, CBFB-MYH11 Nested PCR Result: Positive, breakpoint A, Kit pos", "RUNXT-RUNX1T1 + ckit", "WT1, CKIT mutations",

"c-kit mut Exon 8 and 17; RUNX1/RUNXT1", "c-kit neg, RUNX1-RUNX1T1 + c-kit", "c-kit pos")

then molecular_rev="c kit";

else if molecular in ("NPM1 neg, FLT3-ITD neg, FLT-3 TKD neg", "Absent", "Asn822Lys mutation in exon 17 of the KIT gene, AML-ETO fusion transcripts 3.6 logs reduction",

"BCR-ABL Neg, CRLF2 rearrangement", "BCR-ABL Neg, JAK2 neg, CALR neg, SRSF2 mutation", "BCR-ABL neg", "BCR-ABL negative, KMT2a negative","BCR-ABL1 neg",

"BCR/ABL neg", "BCR/ABL neg, JAK 2 neg", "BCR/ABL neg, JAK2 neg", "BCR/ABL1 neg", "BCR/ABL1 neg,", "BCR/ABL1 neg, CSF neg,",

"DNMT3A pos, IDH1 pos, FLT3-ITD neg, PNH neg", "ETV6-ABL1, ABL1 signal present on der(12)", "FLT3-ITD ; NPM negative","FLT3-ITD neg, FLT3-TKD neg, NPM1 neg",

"FLT3-ITD neg, FLT3-TKD neg, NPM1 neg, BCOR, DNMT3A, IDH2, KRAS, STAT2, TET2 mutations", "FLT3-ITD neg, FLT3-TKD neg, NPM1 neg, CUX1 mutation",

"FLT3-ITD neg, FLT3-TKD neg, NPM1 neg, DNM T3a, IDH, CEBPA, SRSF2 mutations", "FLT3-ITD neg, FLT3-TKD neg, NPM1 neg, DNMT3A, IDH1, PHF6 mutations",

"FLT3-ITD neg, FLT3-TKD neg, NPM1 neg, GNAS, IDH2, SRSF2, PHF6 mutations", "FLT3-ITD neg, FLT3-TKD neg, NPM1 neg, GNAS, IDH2, SRSF2, PHF6 mutations",

"FLT3-ITD neg, FLT3-TKD neg, NPM1 neg, IDH1, DNMT3A mutations", "FLT3-ITD neg, FLT3-TKD neg, NPM1 neg, IDH2, KMT2A mutations",

"FLT3-ITD neg, FLT3-TKD neg, NPM1 neg, NRAS, SH2B3 mutations", "FLT3-ITD neg, FLT3-TKD neg, NPM1 neg, SRSF2, DDX41 mutations", "FLT3-ITD neg, NPM1 neg",

"FLT3-ITD negative, FLT3-TKD negative, NPM1 negative", "JAK 2 neg, CALR neg", "JAK-2 neg", "JAK2 V617F neg, CALR neg",

"JAK2 V617F neg, JAK2 exon 12 neg, CALR neg", "JAK2 and CLAR neg", "JAK2 neg", "JAK2 neg, BCR/ABL neg", "JAK2 neg, BCR/ABL neg, CALR neg",

"JAK2 neg, CALR Neg", "JAK2NEG CALRNEG", "Jak 2 neg", "Jak 2 neg, BCR/ABK neg", "MLL", "MLL rearragement 94%", "Molecular unsuccessful",

"Mutation Phe359Val , TKI resistance; BCR-ABL", "NA", "NEG", "NPM1 Neg; Flt3-ITD neg; Flt3-TKD neg", "NPM1 Negative","NPM1 incon, FLT3-ITD incon, FLT3-TKD neg",

"NPM1 inconclusive, FLT3 neg", "NPM1 neg, FLT3-ITD neg, FLT-3 TKD neg", "NPM1 neg, FLT3-ITD neg", "NPM1 neg, FLT3-ITD neg, FLT3-TKD neg",

"NPM1 neg, FLT3-ITD neg, FLT3-TKD neg, CEBPA failed", "NPM1 neg, FLT3-ITD neg, FLT3-TKD neg, IDH1, JAK2, SH2B3, SRSF2 mutations",

"NPM1 neg, FLT3-ITD neg, FLT3-TKD neg, IDH2 and DMNT3A mutations", "NPM1 neg, FLT3-ITD neg, FLT3-TKD neg, IDH2, RUNX1, SETBP1, TET2, EZH2 mutations",

"NPM1 neg, FLT3-ITD neg, FLT3-TKD neg, KMT2A neg, CEBPA neg", "NPM1 neg, FLT3-ITD neg, FLT3-TKD neg, KMT2A rearrangement",

"NPM1 neg, FLT3-ITD neg, FLT3-TKD neg, NRAS mutation", "NPM1 neg, FLT3-ITD neg, FLT3-TKD neg, PTPN11 mutation",

"NPM1 neg, FLT3-ITD neg, FLT3-TKD neg, RUNX1/RUNX1T1 neg", "NPM1 neg, FLT3-ITD neg, KMT2A", "NPM1 neg, FLt3-ITD neg", "NPM1inconcl, FLT3-ITD neg, FLT3-TKD neg",

"Neg", "RAEB-2", "SRSF2, IDH, CEBPA mutations", "c-kit neg", "del(11q), del(13q)", "neg", "neg for del 11q,13q,17p, and trisomy 12",

"no MLL rearrangement", "pending", "")

then molecular_rev="neg";

else if molecular in ("NPM1 neg, FLT3-ITD neg, FLT-3 TKD pos","NPM1 neg, FLT3-ITD pos (low), FLT-3 TKD neg", "FLT-ITD and NPM1",

"FLT-ITD pos, FLT3-TKD neg, NPM1 pos, NPM1 and FLT3-ITD mutations", "FLT3 ITD Pos, FLT3 TKD Neg, NPM1 Neg, KMT2A Pos",

"FLT3 ITD pos, NPM1 pos, FLT3 TKD neg, SS3B1 and WT1 mutations", "FLT3-ITD", "FLT3-ITD low", "FLT3-ITD neg, FLT3-TKD pos, NPM1 neg, CBL, FLT3, RUNX1 (2 mutations), SF3B1 mutations",

"FLT3-ITD neg, FLT3-TKD pos, NPM1 neg, FLT3, KIT mutations", "FLT3-ITD pos", "FLT3-ITD pos, FLT3-ITD, NPM1, NRAS, DNMT3a mutations",

"FLT3-ITD pos, FLT3-TKD neg, NPM1 neg, RUNX1/RUNX1T1 neg, DNMT3A, IDH1 BCOR mutations", "FLT3-ITD pos, FLT3-TKD neg, NPM1 pos, DNMT3A, KRAS, NPM1 BCOR mutations",

"FLT3-ITD pos, FLT3-TKD neg, NPM1 pos, FLT3, DNMT3A, NPM1 mutations", "FLT3-ITD pos, FLT3-TKD neg, NPM1 pos, IDH2, SRSF mutations",

"FLT3-ITD pos, FLT3-TKD neg, NPM1 pos, NPM1, FLT3-ITD, DNMT3 mutations", "FLT3-ITD, NPM1", "FLT3-TKD, NPM1","NPM1 Neg, Flt3-ITD Pos, Flt3-TKD Neg",

"NPM1 Pos, FLTD -ITD positive; FLTD-D835 Negative", "NPM1 Pos, FlLT3-ITD pos, Flt-3-TKD neg", "NPM1 inconc; Flt3-ITD pos (int); Flt3-TKD neg",

"NPM1 neg, FLT-3 ITD pos low level, FLT-3 TDK neg", "NPM1 neg, FLT3-ITD int pos (25.9%), FLT3-TKD neg", "NPM1 neg, FLT3-ITD low pos, FLT#-TKD neg",

"NPM1 neg, FLT3-ITD neg, FLT3-TKD pos, BCR/ABL1 neg, FLT3, U2Af1, WT1 mutations", "NPM1 neg, FLT3-ITD neg, FLT3-TKD pos, RUNX1, RAD21 and FLT3 mutations",

"NPM1 neg, FLT3-ITD pos (Hight)", "NPM1 neg, FLT3-ITD pos (low)", "NPM1 neg, FLT3-ITD pos int level, FLT3-TKD neg", "NPM1 neg; Flt3-ITD pos (high)",

"NPM1 neg; Flt3-ITD pos (int), Flt3-TKD neg", "NPM1 pos, FLT-3 pos (High), FLT3-ITD", "NPM1 pos, FLT3 pos (high)", "NPM1 pos, FLT3 pos Intermediate",

"NPM1 pos, FLT3 pos high", "NPM1 pos, FLT3 pos intermediate", "NPM1 pos, FLT3 poslow, NPM1 and FLT3-ITD", "NPM1 pos, FLT3-ITD",

"NPM1 pos, FLT3 ITD pos (low levels), ITD-TKD neg", "NPM1 pos, FLT3 pos (high)", "NPM1 pos, FLT3 pos Intermediate", "NPM1 pos, FLT3 pos high",

"NPM1 pos, FLT3 pos intermediate", "NPM1 pos, FLT3 poslow, NPM1 and FLT3-ITD", "NPM1 pos, FLT3-ITD", "NPM1 pos, FLT3-ITD int pos, FLT3-TKD neg",

"NPM1 pos, FLT3-ITD neg, FLT3-TKD pos", "NPM1 pos, FLT3-ITD pos", "NPM1 pos, FLT3-ITD pos (high), FLT3-TKD neg)", "NPM1 pos, FLT3-ITD pos (int)",

"NPM1 pos, FLT3-ITD pos (intermed level), FLT3-TKD neg)", "NPM1 pos, FLT3-ITD pos (inty level), FLT3-TKD neg", "NPM1 pos, FLT3-ITD pos int level, FLT3-TKD neg",

"NPM1 pos, FLT3-ITD pos, FLT3 TKD -ve", "NPM1 pos, FLT3-ITD pos, FLT3-TKD neg", "NPM1 pos, FLT3-ITD pos, FLT3-TKD neg, DNMT3A, NPM1, TET2, FLT3, JAK1 mutations",

"NPM1 pos, FLT3-ITD pos, FLT3-TKD neg, FLT3, ASXL1, NPM1 mutations", "NPM1 pos, FLT3-ITD pos, MLL", "NPM1 pos, Flt3-ITD pos (low); Flt3-TKD neg",

"NPM1 pos; Flt3-ITD pos (int); Flt3-TKD neg")

then molecular_rev="flt3";

else if molecular in ("ASXL1, TET2, DNMT3A", "FLT3-ITD neg, FLT3-TKD neg, NPM1 neg, KMT2A neg, ASXL1, BCOR, CEPB, SRSF, STAG2, TET2 mutations",

"FLT3-ITD neg, FLT3-TKD neg, NPM1 neg, SRSF2, ASXL1, TET2, TP53, NRAS mutations","FLT3-ITD neg, FLT3-TKD neg, NPM1 pos, ASXL1, RAD21, SRSF2, TET2 mutations",

"Jak 2 pos, ASXL1 pos", "Jak2 neg CALR pos, ASXL1 pos", "NPM1 neg, FLT3-ITD neg, FLT3-TKD neg, ASXL1 mutation",

"NPM1 neg, FLT3-ITD neg, FLT3-TKD neg, KIT 25%; CEBPA 80%; WT1 40%, ASXL1 51%", "NPM1 pos; Flt3-ITD neg, ASXL1 pos, DNMT 3 pos, CE$BPA 1 al pos")

then molecular_rev="asxl1";

else if molecular in ("BCR-ABL", "BCR-ABL (p210) pos", "BCR-ABL pos", "BCR-ABL t(9;22)","BCR-ABL1 pos", "BCR-ABL1+", "BCR/ABL Positive", "BCR/ABL pos", "BCR/ABL pos (3.0 log)",

"BCR/ABL pos (4.5 log), NPM1 pos", "BCR/ABL pos (4.9 log)", "BCR/ABL1 pos", "Jak 2 neg, BCR/ABL,Fibrosis", "NPM1 neg, FLT3-ITD neg, BCR/ABL",

"Philadelphia +ve, BCR-ABL", "T315I mutation, BCR-ABL")

then molecular_rev="bcr abl";

else if molecular in ("BCR-ABL neg, JAK2 V617 F mutation pos", "BCR/ABL1 neg, JAK2 V617F pos, CALR neg, JAK2 exon 12",'JAK 2 pos', "JAK-2 pos", "JAK2", "JAK2 Pos",

"JAK2 V617F mutation", "JAK2 V617F pos, CALR neg, JAK2 exon 12 neg, SF3B1 and TET2 mutations", "JAK2 pos", "JAK2 pos, CALR neg", "JAK2+",

"JAK2, V617F mutations", "Jak 2 Positive", "Jak 2 pos", "Jak 2 pos, CALR neg", "Jak-2 pos", "NPM1 Neg; Flt3-ITD neg; Flt3-TKD neg; JAK 2 pos",

"NPM1 neg, FLT3-ITD neg, JAK2 pos, RUNX1 pos, DNMT3A pos, SRSF2 pos, TET2 pos, CEBPA pos", "Post-ET JAK2 pos" )

then molecular_rev="jak2";

else if molecular in ("BCR/ABL neg, TP53", 'FLT3-ITD neg, FLT3-TKD neg, NPM1 neg, TP53, BCOR, mutations.', 'JAK 2 neg, TP53', "NPM1 neg, FLT3-ITD neg, TP53 mutation",

"TP53 mutation", "TP53, RUNX1, SF3B1 mutation", "tp53" )

then molecular_rev="tp53";

else if molecular in ("BCR/ABL1 neg, FLT3, RUNX1, SRSF2, TET2, EZH2 mutations", "FLT3-ITD neg, FLT3-TKD neg, NPM1 neg, NRAS, PPM1D, TET2, DNMT3A, RUNX1 mutations",

"FLT3-ITD neg, FLT3-TKD neg, NPM1 neg, PML/RARA neg, KMT2A, 11q23 Rearrangement neg, RUNX1/RUNX1T1 neg, IDH1 pos, NRAS pos",

"FLT3-ITD neg, FLT3-TKD neg, NPM1 neg, RUNX1, SF3B1 mutations", "KIT neg, RUNX1/RUNX1T1 mutations", "NPM1 neg, FLT3-ITD neg, FLT3-TKD neg, RUNX1 mutation",

"NPM1 neg, FLT3-ITD neg, KIT neg, CBFB-MYH11 Nested PCR pos, CBFB-MYH11 RQ-PCR pos, class II NRAS, class IIIA RUNX1 , class IIIA, FLT3-TKD and class IV KDM6A mutations.",

"NPM1 neg, FLT3-ITD pos, FLT3-TKD neg, RUNX1 pos, WT1, NRAS mutations", "RUNX1 pos, SRSF2 pos" )

then molecular_rev="runx";

else if molecular in ("CALCR pos, SRSF-2 mutation, ETV6 pos", "CALR pos, JAK2 neg", "JAK2 neg, CALR pos", "NPM1 neg, FLT3-ITD neg, JAK 2 neg, CALR pos" )

then molecular_rev="calr";

else if molecular in ("CBFB-MYH11", "FLT3-ITD neg, FLT3-TKD neg, NPM1 neg, CBL, AF mutations", "FLT3-ITD neg, NPM1 neg, KIT neg, CBFB-MYH11 mutations",

"FLT3-ITD neg, NPM1 neg, KIT neg, CBFB-MYH11 mutations" )

then molecular_rev="cbf";

else if molecular in ("FLT3-ITD neg, FLT3-TKD inconclusive, NPM1 pos, CEBPA, DNMT3A, FLT3, IDH1, NPM1, NRAS mutations",

"FLT3-ITD neg, FLT3-TKD neg, NPM1 pos, CEBPA, IDH1, NPM1 mutations","FLT3-ITD neg, NPM1 pos, IDH1, NPM1, CUX1 mutations","NPM1 Pos, FLT-3 ITD pos, FLT-3 TKD neg",

"NPM1 pos", "NPM1 pos, FLT3-ITD neg, FLT-3 TKD neg", "NPM1 pos, FLT-3 ITD pos", "NPM1 pos, FLT3 ITD pos (low levels), ITD-TKD neg",

"NPM1 pos, FLT3-ITD neg", "NPM1 pos, FLT3-ITD neg, FLT3-TKD neg", "NPM1 pos, FLT3-ITD neg, FLT3-TKD neg, DNMT3A, FLT3, NPM1 mutationsV",

"NPM1 pos, FLT3-ITD neg, FLT3-TKD neg, DNMT3A, NPM1, NRAS, PHF6, CBL mutations", "NPM1 pos, FLT3-itd neg, FLT3-TKD pos", "NPM1 pos; Flt3-ITD neg",

"NPM1 pos; Flt3-ITD neg; Flt3-TKD neg", "NPM1 pos; Flt3-ITD neg; Flt3-TKD neg, NPM1 mutation" )

then molecular_rev="npm1";

else if molecular in ("IDH2 mutation, MLL ITD", "KMT2A, MLL", "KMT2A-MLLT3 mutation", "MLL rearrangement", "MLL rearrangement (94%)", "MLL/AF4 pos", "MLLT3-MLL" )

then molecular_rev="mll";

else if study_id ="XXXXX"

then molecular_rev="pml rara";

**run**;

**data** master_opioid7_0;

set master_opioid6;

length diagnose_rev $100.;

if diagnose in ("ALL ","ALl ", "MPAL ") then diagnose_rev="ALL/MPAL";

else if diagnose in ("AML ") then diagnose_rev="AML";

else if diagnose in ("CLL ","Lymphoma ") then diagnose_rev="AML";

else if diagnose in ("CML ","MF ","MPN ") then diagnose_rev="CML/MF/MPN";

else if diagnose in ("SAA") then diagnose_rev="SAA";

else if diagnose in ("DCN ", "GATA2 Deficiency", "Non-Malign ") then diagnose_rev="Others (DCN/GATA2 Deficiency/Non-Malign";

else if diagnose="" then diagnose_rev="";

length donor_rev $100.;

if donor="Haplo" then donor_rev="Haplo";

else if donor in ("MRD","Syngeneic") then donor_rev="MRD";

else if donor in ("URD") then donor_rev="URD";

length KPS_rev $100.;

if kps_prior_sct in (**90**,**100**) then KPS_rev="90-100";

else if kps_prior_sct in (**60**,**70**,**80**) then KPS_rev="60-80";

length hct_ci_rev $100.;

if hct_ci in (**0**,**1**,**2**) then HCT_CI_rev="0-2";

else if hct_ci>=**3** then HCT_CI_rev="3 or more";

length dri_rev $100.;

if dri_0_low___1_intermediate_2_hi in ("0","1") then DRI_rev="0-1";

else if dri_0_low___1_intermediate_2_hi in ("2","3") then DRI_rev="2-3";

label therapy_related_rev="Therapy";

label diagnose_rev="Diagnosis";

label donor_rev="Donor";

label stage_rev="Stage";

if source in ("PB", "PBSC") then source_rev="PB";

else if source in ("BM") then source_rev="BM";

label source_rev="Source";

length molecular_rev2 $100.;

if molecular_rev in ("neg") then molecular_rev2="Neg";

else if molecular_rev ne "" then molecular_rev2="Others";

label molecular_rev2="Molecular";

label cyto_code="Cytogenetics";

label KPS_rev="KPS";

label HCT_CI_rev="HCT-CI";

label DRI_rev="DRI";

*label aGVH_grade_rev="aGVHD";

label cGVHD_grade_rev_3="cGVHD";

if cyto_code in (**0**,**1**) then cyto_code2="0-1";

else if cyto_code=**2** then cyto_code2="2";

**run**;

**data** master_opioid7_1;

set master_opioid7_0;

length stage_rev2 $100.;

if stage_rev in ("CR1") then stage_rev2="CR1";

else if stage_rev in ("CR2","CR3","Partial response") then stage_rev2="Others";

/*** remove patients with non-malignant diagnoses ***/

length diagnose_final $100.;

if diagnose in ("ALL", "ALl", "MPAL") then diagnose_final="ALL/MPAL";

else if diagnose in ("AML") then diagnose_final="AML";

else if diagnose in ("CML", "CLL", "Lymphoma") then diagnose_final="Other(CML/CLL/Lymphoma)";

else if diagnose in ("CMML", "MDS") then diagnose_final="CMML/MDS";

**run**;

**data** master_opioid7;

set master_opioid7_1;

length category $100.;

if user="Chronic User" and grouping="Before" then category="Chronic user who started before BMT";

else if user="Chronic User" and grouping="After" then category="Chronic user who started after BMT";

else if user="Non chronic user" and grouping="After" then category="Non-chronic user who started after BMT";

else if user="Non chronic user" and grouping="Before" then category="Non-chronic user who started before BMT";

else category="Non opioid user";

if indexyear in (**2010**,**2011**,**2012**,**2013**,**2014**,**2015**) then index_year="2010-2015";

else if indexyear in (**2016**,**2017**,**2018**,**2019**) then index_year="2016-2019";

if earliest_start<**0** and time_cGVHD=**.** then start_after_cGVHD=**.**;

else if earliest_start<**0** and time_cGVHD>**.** then start_after_cGVHD=**0**;

else if earliest_start>=**0** and earliest_start<time_cGVHD then start_after_cGVHD=**0**;

else if earliest_start>=**0** and earliest_start>=time_cGVHD and time_cGVHD>**.** then start_after_cGVHD=**1**;

else if earliest_start>=**0** and earliest_start>=time_cGVHD and time_cGVHD=**.** then start_after_cGVHD=**.**;

if earliest_start<**0** and time_aGVHD=**.** then start_after_aGVHD=**.**;

else if earliest_start<**0** and time_aGVHD>**.** then start_after_aGVHD=**0**;

else if earliest_start>=**0** and earliest_start<time_aGVHD then start_after_aGVHD=**0**;

else if earliest_start>=**0** and earliest_start>=time_aGVHD and time_aGVHD>**.** then start_after_aGVHD=**1**;

else if earliest_start>=**0** and earliest_start>=time_aGVHD and time_aGVHD=**.** then start_after_aGVHD=**.**;

if earliest_start<**0** and time_aGVHD24=**.** then start_after_aGVHD24=**.**;

else if earliest_start<**0** and time_aGVHD24>**.** then start_after_aGVHD24=**0**;

else if earliest_start>=**0** and earliest_start<time_aGVHD24 then start_after_aGVHD24=**0**;

else if earliest_start>=**0** and earliest_start>=time_aGVHD24 and time_aGVHD24>**.** then start_after_aGVHD24=**1**;

else if earliest_start>=**0** and earliest_start>=time_aGVHD24 and time_aGVHD24=**.** then start_after_aGVHD24=**.**;

**run**;

**data** master_opioid7_rev;

set master_opioid7;

if find(acvhd_organ_involvement3,'gut')>**0** or find(acvhd_organ_involvement3,'Gut')>**0** or find(acvhd_organ_involvement3,'lower GI')>**0** then aGVHD_gut_related=**1**;

if find(cgvhd_organ3,'gut')>**0** or find(cgvhd_organ3,'Gut')>**0** or find(cgvhd_organ3,'lower GI')>**0** then cGVHD_gut_related=**1**;

if find(acvhd_organ_involvement3,'gut')>**0** and aGVHD=**1** then aGVHD_gut=**1**;

else if find(acvhd_organ_involvement3,'Gut')>**0** and aGVHD=**1** then aGVHD_gut=**1**;

else if find(acvhd_organ_involvement3,'lower GI')>**0** and aGVHD=**1** then aGVHD_gut=**1**;

else aGVHD_gut=**0**;

if find(cgvhd_organ3,'gut')>**0** and cGVHD=**1** then cGVHD_gut=**1**;

else if find(cgvhd_organ3,'Gut')>**0** and cGVHD=**1** then cGVHD_gut=**1**;

else if find(cgvhd_organ3,'lower GI')>**0** and cGVHD=**1** then cGVHD_gut=**1**;

else cGVHD_gut=**0**;

if find(cause_of_death,'gut')>**0** or find(cause_of_death,'Gut')>**0** or find(cause_of_death,'lower GI')>**0** then COD_gut=**1**;

else if cause_of_death ne "" and find(cause_of_death,'gut', 'Gut', 'lower GI')=**0** then COD_gut=**0**;

if total_days>=**30** then total_days_above_30days=**1**;

else if total_days<**30** and total_days>**.** then total_days_above_30days=**0**;

if total_days>=**10** then total_days_above_10days=**1**;

else if total_days<**10** and total_days>**.** then total_days_above_10days=**0**;

**run**;

**data** descriptive; set master_opioid7_rev;

label days_to_last_f_u3="Time to Last Follow Up";

label relapse_days="Time to Relapse";

label RFS_days="Time to Relapse or Death";

label aGVHD_days="Time to aGVHD";

label cGVHD_days="Time to cGVHD";

label GRFS_days="Time to Graft Failure or Relapse or Death";

label NRM_days="Time to Non-relapse Related Mortality";

if user="Opioid user" then user_2grp=**1**;

else if user="Non opioid user" then user_2grp=**0**;

if aGVHD_Days>**100** then do; aGVHD_days2=**100**; aGVHD2=**0**; end;

else do aGVHD_days2=aGVHD_Days; aGVHD2=aGVHD; end;

if aGVHD2=**2** then aGVHD3=**0**;

else aGVHD3=aGVHD2;

if aGVHD24_Days>**100** then do; aGVHD24_days2=**100**; aGVHD24_2=**0**; end;

else do aGVHD24_days2=aGVHD24_Days; aGVHD24_2=aGVHD24; end;

if aGVHD24_2=**2** then aGVHD24_3=**0**;

else aGVHD24_3=aGVHD24_2;

if NRM=**2** then NRM2=**0**;

else NRM2=NRM;

length donor_group $100.;

if find(donor_type, "Haplo")>**0** or find(donor_type, "HAPLO")>**0** then donor_group="MM (Haplo or MM URD)";

else if donor_type in ("MSF","MSM","Syngeneic") then donor_group="MRD";

else if donor_type in ("MUD","MUD- M","MUD-F","MUD-M") and find(hla_match_0001,"9/10")>**0** then donor_group="MM (Haplo or MM URD)";

else if donor_type in ("MUD","MUD- M","MUD-F","MUD-M") and find(hla_match_0001,"9/10")<=**0** then donor_group="MUD";

length molecular_rev2 $100.;

if molecular_rev="neg" then molecular_rev2="Neg";

else if molecular_rev ne "" then molecular_rev2="Muted";

else if molecular_rev="" then molecular_rev2="";

**run**;

**data** cGVHD;

set descriptive;

if relapse_days<**100** then delete;

if dead=**1** and days_to_last_f_u3<**100** then delete;

if cGVHD=**2** then cGVHD2=**0**;

else cGVHD2=cGVHD;

if start_after_cGVHD=**1** then do; cGVHD_final=**.**; cGVHD_days_final=**.**; end;

else do; cGVHD_final=cGVHD2; cGVHD_days_final=cGVHD_days; end;

if start_after_aGVHD=**1** then do; aGVHD_final=**.**; aGVHD_days_final=**.**; end;

else do; aGVHD_final=aGVHD2;aGVHD_days_final=aGVHD_days2; end;

/** GVHD free, relapse free survival (GRFS) ***/

if dead=**1** or relapse3=**1** or cGVHD_final=**1** or aGVHD_final=**1**

then do; GRFS=**1**;

GRFS_days=min(days_to_last_f_u3, days_to_relapse_date3, days_to_agvhd34_date3, days_to_cGVHD_mod_sev_date3); end;

else do; GRFS=**0**; GRFS_days=days_to_last_f_u3; end;

**run**;

**data** aGVHD;

set descriptive;

if aGVHD_Days>**100** then do; aGVHD_days2=**100**; aGVHD2=**0**; end;

else do aGVHD_days2=aGVHD_Days; aGVHD2=aGVHD; end;

if start_after_aGVHD=**1** then do; aGVHD_final=**.**; aGVHD_days_final=**.**; end;

else do; aGVHD_final=aGVHD2;aGVHD_days_final=aGVHD_days2; end;

if start_after_aGVHD24=**1** then do; aGVHD24_final=**.**;aGVHD24_days_final=**.**; end;

else do; aGVHD24_final=aGVHD24_2;aGVHD24_days_final=aGVHD24_days2; end;

**run**;

/*********************************************************/

/********* compare opioid vs non-opioid group ************/

/*********************************************************/

**proc** **freq** data=descriptive; tables dead relapse_ind aGVHD cGVHD NRM RFS GRFS; **run**;

**proc** **freq** data=descriptive; tables user_final3*dead/chisq fisher; **run**;

**proc** **freq** data=descriptive; tables user_final3*relapse/chisq fisher; **run**;

**proc** **freq** data=descriptive; tables user_final3*RFS/chisq fisher; **run**;

**proc** **freq** data=descriptive; tables user_final3*aGVHD/chisq fisher; **run**;

**proc** **freq** data=descriptive; tables user_final3*NRM/chisq fisher; **run**;

**proc** **freq** data=cGVHD; tables user_final3*cGVHD/chisq fisher; **run**;

**proc** **freq** data=cGVHD; tables user_final3*GRFS/chisq fisher; **run**;

**proc** **freq** data=descriptive; tables user_final3/chisq fisher; **run**;

**proc** **contents** data=master_opioid; **run**;

**proc** **freq** data=master_opioid; tables ethnicdiv_q_da; **run**;

**proc** **ttest** data=descriptive; class user_final3; var days_to_discon_of_immuno; **run**;

**proc** **means** data=descriptive median min max; class user_final3; var days_to_discon_of_immuno; **run**;

**proc** **npar1way** data=descriptive wilcoxon; class user_final3; var days_to_discon_of_immuno; **run**;

**proc** **means** data=descriptive n mean std median min max; where user_final3="Opioid user"; var earliest_start; **run**;

**data** descriptive; set descriptive; label user_final3="Opioid User (Yes vs No)"; **run**;

/**********************************************************************************************/

/********************** Univariable and Multivariable model ***********************************/

/**********************************************************************************************/

**proc** **freq** data=cGVHD; tables cGVHD_gut; **run**;

**proc** **freq** data=descriptive; tables aGVHD_gut; **run**;

**proc** **freq** data=descriptive; tables donor_rev; **run**;

title "Univariate analysis for OS";

%***UNI_PHREG***(dataset=descriptive, event=days_to_last_f_u3, censor=dead,

clist=user_final3(ref="Non opioid user")*age_group(ref="60+")*diagnose_final(ref="AML")*donor_group(ref="MUD")*gender(ref="Female")

*stage_rev2(ref="Others")*source_rev(ref="PB")*RIC_MAC(ref="RIC")*molecular_rev2(ref="Others")*cyto_code2(ref="0-1")*KPS_rev(ref="60-80")*hct_ci_rev(ref="0-2")

*dri_rev(ref="0-1"),

nlist=,

logrank=F,

type3=T,

outpath=X:\xxxxx\Output for Vetting\,

fname=OS UVA Oct **31**);

title;

/************** select variables with p<0.1 from UVA into MVA, retain variables withh p<0.05 *******************/

**proc** **phreg** data=descriptive;

class user_final3(ref="Non opioid user") age_group(ref="60+") diagnose_final(ref="AML") donor_group(ref="MUD") molecular_rev2(ref="Others")

cyto_code2(ref="0-1") KPS_rev(ref="60-80") hct_ci_rev(ref="0-2") dri_rev(ref="0-1") ;

model days_to_last_f_u3*dead(**0**)=user_final3 age_group diagnose_final donor_group molecular_rev2 cyto_code2 KPS_rev hct_ci_rev dri_rev

/selection=backward slstay=**0.05**;

**run**;

**proc** **phreg** data=descriptive;

class user_final3(ref="Non opioid user") age_group(ref="60+") donor_group(ref="MUD") cyto_code2(ref="0-1") hct_ci_rev(ref="0-2") ;

model days_to_last_f_u3*dead(**0**)=user_final3 age_group donor_group cyto_code2 hct_ci_rev ;

hazardratio user_final3/diff=pairwise;

hazardratio age_group/diff=pairwise;

hazardratio donor_group/diff=pairwise;

hazardratio cyto_code2/diff=pairwise;

hazardratio hct_ci_rev/diff=pairwise;

**run**;

/*************************************************************/

/********************** RFS ***********************************/

/*************************************************************/

title "Univariate analysis for RFS";

%***UNI_PHREG***(dataset=descriptive, event=RFS_days, censor=RFS,

clist=user_final3(ref="Non opioid user")*age_group(ref="60+")*diagnose_final(ref="AML")*donor_group(ref="MUD")*gender(ref="Female")

*stage_rev2(ref="Others")*source_rev(ref="PB")*RIC_MAC(ref="RIC")*molecular_rev2(ref="Others")*cyto_code2(ref="0-1")*KPS_rev(ref="60-80")*hct_ci_rev(ref="0-2")

*dri_rev(ref="0-1"),

nlist=,

logrank=F,

type3=T,

outpath=X:\xxxxx\Output for Vetting\,

fname=RFS UVA Oct **31**);

title;

/************** select variables with p<0.1 from UVA into MVA, retain variables withh p<0.05 *******************/

**proc** **phreg** data=descriptive;

class user_final3(ref="Non opioid user") age_group(ref="60+") diagnose_final(ref="AML") donor_group(ref="MUD") cyto_code2(ref="0-1") KPS_rev(ref="60-80")

hct_ci_rev(ref="0-2") dri_rev(ref="0-1") ;

model RFS_days*RFS(**0**)=user_final3 age_group diagnose_final donor_group cyto_code2 KPS_rev hct_ci_rev dri_rev

/selection=backward slstay=**0.05**;

**run**;

**proc** **phreg** data=descriptive;

class user_final3(ref="Non opioid user") age_group(ref="60+") donor_group(ref="MUD") cyto_code2(ref="0-1") /*hct_ci_rev(ref="0-2")*/ dri_rev(ref="0-1") ;

model RFS_days*RFS(**0**)=user_final3 age_group donor_group cyto_code2 /*hct_ci_rev*/ dri_rev ;

hazardratio user_final3/diff=pairwise;

hazardratio age_group/diff=pairwise;

hazardratio donor_group/diff=pairwise;

hazardratio cyto_code2/diff=pairwise;

*hazardratio hct_ci_rev/diff=pairwise;

hazardratio dri_rev/diff=pairwise;

**run**;

/*************************************************************/

/********************** GRFS ***********************************/

/*************************************************************/

title "Univariate analysis for GRFS";

%***UNI_PHREG***(dataset=cGVHD, event=GRFS_days, censor=GRFS,

clist=user_final3(ref="Non opioid user")*age_group(ref="60+")*diagnose_final(ref="AML")*donor_group(ref="MUD")*gender(ref="Female")

*stage_rev2(ref="Others")*source_rev(ref="PB")*RIC_MAC(ref="RIC")*molecular_rev2(ref="Neg")*cyto_code2(ref="0-1")*KPS_rev(ref="60-80")*hct_ci_rev(ref="0-2")

*dri_rev(ref="0-1"),

nlist=,

logrank=F,

type3=T,

outpath=X:\xxxxx\Output for Vetting\,

fname=GRFS UVA Oct **31**);

title;

**proc** **freq** data=cGVHD; tables molecular_rev2; **run**;

/************** select variables with p<0.1 from UVA into MVA, retain variables withh p<0.05 *******************/

**proc** **phreg** data=cGVHD;

class user_final3(ref="Non opioid user") donor_group(ref="MUD") RIC_MAC(ref="RIC") dri_rev(ref="0-1");

model GRFS_days*GRFS(**0**)=user_final3 donor_group RIC_MAC dri_rev /selection=backward slstay=**0.05**;

**run**;

title "backward selection for GRFS";

%***FineGray_Sel***(dsn=cGVHD,event=GRFS_days, censor=GRFS,

var=user_final3 donor_group RIC_MAC cyto_code2 dri_rev,

cvar=user_final3(ref="Non opioid user")*donor_group(ref="MUD")*RIC_MAC(ref="RIC")*cyto_code2(ref="0-1")*dri_rev(ref="0-1"),

alpha=**0.05**,

inc=**1**,

type3=T,

outpath=X:\xxxxx\Output for Vetting\,

filename=GRFS Backward selection);

title;

**proc** **phreg** data=cGVHD;

class user_final3(ref="Non opioid user") donor_group(ref="MUD") dri_rev(ref="0-1");

model GRFS_days*GRFS(**0**)=user_final3 donor_group dri_rev;

hazardratio user_final3/diff=pairwise;

hazardratio donor_group/diff=pairwise;

hazardratio dri_rev/diff=pairwise;

**run**;

/*************************************************************/

/********************** relapse *****************************/

/*************************************************************/

title "Univariate analysis for relapse";

%***UNI_PHREG***(dataset=descriptive, event=relapse_days, censor=relapse_ind, eventcode=**1**,

clist=user_final3(ref="Non opioid user")*age_group(ref="60+")*diagnose_final(ref="AML")*donor_group(ref="MUD")*gender(ref="Female")

*stage_rev2(ref="Others")*source_rev(ref="PB")*RIC_MAC(ref="RIC")*molecular_rev2(ref="Others")*cyto_code2(ref="0-1")*KPS_rev(ref="60-80")*hct_ci_rev(ref="0-2")

*dri_rev(ref="0-1"),

nlist=,

logrank=F,

type3=T,

outpath=X:\xxxxx\Output for Vetting\,

fname=Relapse UVA Oct **31**);

title;

title "backward selection for relapse";

%***FineGray_Sel***(dsn=descriptive,event=relapse_days, censor=relapse_ind, event_code=**1**,

var=user_final3 stage_rev2 source_rev RIC_MAC cyto_code2 dri_rev,

cvar=user_final3(ref="Non opioid user")*stage_rev2(ref="Others")*source_rev(ref="PB")*RIC_MAC(ref="RIC")*cyto_code2(ref="0-1")*dri_rev(ref="0-1"),

alpha=**0.05**,

inc=**1**,

type3=T,

outpath=X:\xxxxx\Output for Vetting\,

filename=relapse Backward selection);

title;

/*** MVA ***/

**proc** **phreg** data=descriptive;

class user_final3(ref="Non opioid user") stage_rev2(ref="CR1") RIC_MAC(ref="RIC") cyto_code2(ref="0-1") ;

model relapse_days*relapse_ind(**0**)=user_final3 stage_rev2 RIC_MAC cyto_code2 /eventcode=**1** ;

hazardratio user_final3/diff=pairwise;

hazardratio stage_rev2/diff=pairwise;

hazardratio RIC_MAC/diff=pairwise;

hazardratio cyto_code2/diff=pairwise;

**run**;

/*************************************************************/

/*************************** NRM *****************************/

/*************************************************************/

title "Univariate analysis for NRM";

%***UNI_PHREG***(dataset=descriptive, event=NRM_days, censor=NRM, eventcode=**1**,

clist=user_final3(ref="Non opioid user")*age_group(ref="60+")*diagnose_final(ref="AML")*donor_group(ref="MUD")*gender(ref="Female")

*stage_rev2(ref="Others")*source_rev(ref="PB")*RIC_MAC(ref="RIC")*molecular_rev2(ref="Others")*cyto_code2(ref="0-1")*KPS_rev(ref="60-80")*hct_ci_rev(ref="0-2")

*dri_rev(ref="0-1"),

nlist=,

logrank=F,

type3=T,

outpath=X:\xxxxx\Output for Vetting\,

fname=NRM UVA Oct **31**);

title;

title "backward selection for NRM";

%***FineGray_Sel***(dsn=descriptive,event=NRM_days, censor=NRM, event_code=**1**,

var=user_final3 age_group diagnose_final donor_group molecular_rev2 KPS_rev hct_ci_rev dri_rev,

cvar=user_final3(ref="Non opioid user")*age_group(ref="60+")*diagnose_final(ref="AML")*donor_group(ref="MUD")*molecular_rev2(ref="Others")

*KPS_rev(ref="60-80")*hct_ci_rev(ref="0-2")*dri_rev(ref="0-1"),

alpha=**0.05**,

inc=**1**,

type3=T,

outpath=X:\xxxxx\Output for Vetting\,

filename=NRM Backward selection);

title;

/*** MVA ***/

**proc** **phreg** data=descriptive;

class user_final3(ref="Non opioid user") age_group(ref="60+") donor_group(ref="MUD") hct_ci_rev(ref="0-2");

model NRM_days*NRM(**0**)=user_final3 age_group donor_group hct_ci_rev/eventcode=**1** ;

hazardratio user_final3/diff=pairwise;

hazardratio age_group/diff=pairwise;

hazardratio donor_group/diff=pairwise;

hazardratio hct_ci_rev/diff=pairwise;

**run**;

/*************************************************************/

/*************************** aGVHD *****************************/

/*************************************************************/

title "Univariate analysis for aGVHD";

%***UNI_PHREG***(dataset=aGVHD, event=aGVHD_days_final, censor=aGVHD_final, eventcode=**1**,

clist=user_final3(ref="Non opioid user")*age_group(ref="60+")*diagnose_final(ref="AML")*donor_group(ref="MUD")*gender(ref="Female")

*stage_rev2(ref="Others")*source_rev(ref="PB")*RIC_MAC(ref="RIC")*molecular_rev2(ref="Neg")*cyto_code2(ref="0-1")*KPS_rev(ref="60-80")*hct_ci_rev(ref="0-2")

*dri_rev(ref="0-1"),

nlist=,

logrank=F,

type3=T,

outpath=X:\xxxxx\Output for Vetting\,

fname=aGVHD UVA Oct **31**);

title;

**data** modify_aGVHD; set aGVHD;

if study_id="XXXXX" then do; stage_rev2="Partial response"; source_rev="BM"; end; length stage_rev2 $100.;

if stage_rev in ("CR1") then stage_rev2="CR1";

else if stage_rev in ("CR2","CR3","Partial response") then stage_rev2="Others";**run**;

title "backward selection for aGVHD";

%***FineGray_Sel***(dsn=modify_aGVHD,event=aGVHD_days_final, censor=aGVHD_final, event_code=**1**,

var=user_final3 stage_rev2 source_rev RIC_MAC hct_ci_rev ,

cvar=user_final3(ref="Non opioid user")*stage_rev2(ref="Others")*source_rev(ref="PB")*RIC_MAC(ref="RIC")*hct_ci_rev(ref="0-2"),

alpha=**0.05**,

inc=**1**,

type3=T,

outpath=X:\xxxxx\Output for Vetting\,

filename=aGVHD Backward selection);

title;

**proc** **phreg** data=modify_aGVHD; class source_rev(ref="PB");

model aGVHD_days_final*aGVHD_final(**0**)=source_rev/eventcode=**1** ;

hazardratio source_rev/diff=pairwise;

**run**;

/*** MVA ***/

**proc** **freq** data=descriptive_modify_aGVHD; tables aGVHD2*stage_rev2; **run**;

**proc** **freq** data=descriptive_modify_aGVHD; tables aGVHD2*source_rev; **run**;

**proc** **phreg** data= modify_aGVHD;

class user_final3(ref="Non opioid user") hct_ci_rev(ref="0-2") ;

model aGVHD_days_final*aGVHD_final(**0**)=user_final3 hct_ci_rev /eventcode=**1** ;

hazardratio user_final3/diff=pairwise;

hazardratio hct_ci_rev/diff=pairwise;

**run**;

/*************************************************************/

/************************ cGVHD *****************************/

/*************************************************************/

title "Univariate analysis for cGVHD";

%***UNI_PHREG***(dataset=cGVHD, event=cGVHD_days_final, censor=cGVHD_final, eventcode=**1**,

clist=user_final3(ref="Non opioid user")*age_group(ref="60+")*therapy_related_rev(ref="No")*diagnose_final(ref="AML")*donor_group(ref="MUD")*gender(ref="Female")

*stage_rev2(ref="Others")*source_rev(ref="PB")*RIC_MAC(ref="RIC")*molecular_rev2(ref="Neg")*cyto_code2(ref="0-1")*KPS_rev(ref="60-80")*hct_ci_rev(ref="0-2")

*dri_rev(ref="0-1"),

nlist=,

logrank=F,

type3=T,

outpath=X:\xxxxx\Output for Vetting\,

fname=cGVHD UVA Oct **31**);

title;

title "backward selection for cGVHD";

%***FineGray_Sel***(dsn=cGVHD,event=cGVHD_days_final, censor=cGVHD_final, event_code=**1**,

var=user_final3 age_group donor_group RIC_MAC,

cvar=user_final3(ref="Non opioid user")*age_group(ref="60+")*donor_group(ref="MUD")*RIC_MAC(ref="RIC"),

alpha=**0.05**,

inc=**1**,

type3=T,

outpath=X:\xxxxx\Output for Vetting\,

filename=cGVHD Backward selection);

title;

**proc** **phreg** data=cGVHD;

class user_final3(ref="Non opioid user") donor_group(ref="MUD") RIC_MAC(ref="RIC");

model cGVHD_days_final*cGVHD_final(**0**)=user_final3 donor_group RIC_MAC/eventcode=**1** ;

hazardratio user_final3/diff=pairwise;

hazardratio donor_group/diff=pairwise;

hazardratio RIC_MAC/diff=pairwise;

**run**;

/*********************************************************************************/

/**************************** Descriptive Table *********************************/

/*********************************************************************************/

%***table1macro***(dsn=descriptive,

calist=age_Group,

coplist=,

cononplist=,

group=user_final3,

caexcludelist=,

caincludemissing = yes,

copincludemissing = no,

cononpincludemissing = yes,

overall=yes,

missingtop=no,

copmain=mean,

copsupplement=std,

cononpmain=median,

cononpsupplement=range);

**proc** **freq** data=descriptive; tables user_final3; **run**;

**proc** **freq** data=descriptive; tables age_group*user_final3/chisq fisher; **run**;

**proc** **freq** data=descriptive; tables therapy_related_rev*user_final3/chisq fisher; **run**;

**proc** **freq** data=descriptive; tables index_year*user_final3/chisq fisher; **run**;

**proc** **freq** data=descriptive; tables diagnose_final*user_final3/chisq; **run**;

**proc** **freq** data=descriptive; tables donor_group*user_final3/chisq fisher; **run**;

**proc** **freq** data=descriptive; tables gender*user_final3/chisq fisher; **run**;

**proc** **freq** data=descriptive; tables stage*user_final3/chisq; **run**;

**proc** **freq** data=descriptive; tables stage_rev2*user_final3/chisq; **run**;

**proc** **freq** data=descriptive; tables source_rev*user_final3/chisq fisher; **run**;

**proc** **freq** data=descriptive; tables RIC_MAC*user_final3/chisq fisher; **run**;

**proc** **freq** data=descriptive; tables molecular_rev*user_final3/chisq; **run**;

**proc** **freq** data=descriptive; tables molecular_rev2*user_final3/chisq; **run**;

**proc** **freq** data=descriptive; tables cyto_code*user_final3/chisq fisher; **run**;

**proc** **freq** data=descriptive; tables kps_rev*user_final3/chisq fisher; **run**;

**proc** **freq** data=descriptive; tables hct_ci_rev*user_final3/chisq fisher; **run**;

**proc** **freq** data=descriptive; tables dri_rev*user_final3/chisq fisher; **run**;

**proc** **freq** data=descriptive; tables relapse*user_final3/chisq fisher; **run**;

**proc** **freq** data=descriptive; tables aGVHD3*user_final3/chisq fisher; **run**;

**proc** **freq** data=descriptive; tables aGVHD_gut*user_final3/chisq fisher; **run**;

**proc** **freq** data=cGVHD; tables cGVHD_final*user_final3/chisq fisher; **run**;

**proc** **freq** data=cGVHD; tables cGVHD_gut*user_final3/chisq fisher; **run**;

**proc** **freq** data=aGVHD; tables aGVHD_final*user_final3/chisq fisher; **run**;

**proc** **freq** data=aGVHD; tables aGVHD24_final*user_final3/chisq fisher; **run**;

**proc** **freq** data=descriptive; tables dead*user_final3/chisq fisher; **run**;

**proc** **freq** data=descriptive; tables GRFS*user_final3/chisq fisher; **run**;

**proc** **freq** data=descriptive; tables NRM2*user_final3/chisq fisher; **run**;

**proc** **freq** data=descriptive; tables RFS*user_final3/chisq fisher; **run**;

**proc** **freq** data=descriptive; tables donor_type; **run**;

**proc** **means** data=descriptive mean std median min max; class user_final3; var earliest_start; **run**;

/***** condense cause of death for opioid users *****/

**data** opioid_user_yes2; set opioid_user_yes;

length cause_of_death_grouped $100.;

if cause_of_death__primary_ in ("CMV Colitis","Klebsiella pneumonia","Line sepsis (Staph. Aureus)","Sepsis",

"MRSA pneumonia","Pneumonia, St aureus bacteremia","Pulmonary infection (unknown etiology)", "Recurrence/pProgressive Disease; polymicrobial sepsis")

then cause_of_death_grouped="Sepsis";

else if cause_of_death__primary_ in ("Diffuse alveolar hemorrhage","Cardiac arrest","Resp. infection multiple organisms") then cause_of_death_grouped="Toxicity";

else if cause_of_death__primary_ in ("GvHD lungs","Steroid refractory GvHD GI","Steroid-refractory GvHD gut","cGvHD steroid refractory") then cause_of_death_grouped="GVHD";

else if cause_of_death__primary_ in ("Relapse","Relapse, disease progression","T-cell lymphoma unspecified") then cause_of_death_grouped="Relapse";

else if cause_of_death__primary_ in ("Unknown") then cause_of_death_grouped="";

length cause_of_death_grouped2 $100.;

if cause_of_death__primary_ in ("Relapse","Relapse, disease progression","T-cell lymphoma unspecified") then cause_of_death_grouped2="Relapse related mortality";

else if cause_of_death__primary_ ="Unknown" then cause_of_death_grouped2="";

else if cause_of_death__primary_ ne "" then cause_of_death_grouped2="None-Relapse related mortality";

**run**;

**proc** **freq** data=opioid_user_yes2;tables cause_of_death_grouped2; **run**;

**proc** **freq** data=descriptive; tables user_final3*therapy_related_rev; **run**;

**proc** **freq** data=descriptive; tables user_final3*dead; **run**;

/***** condense cause of death for non-opioid users *****/

**proc** **freq** data=opioid_user_no; tables cause_of_death__primary_; **run**;

**data** opioid_user_no_0;

set opioid_user_no; cause_death=strip(cause_of_death__primary_);

**run**;

**data** opioid_user_no2;

set opioid_user_no_0;

length cause_of_death_grouped $100.;

if cause_death in ("Relapse","Secondary malignancy (AML)","Progession to AML",

"Relapse, multi organ failure secondary to refractory AML","Relapse, pulmonary organ failure","Relapse- blast crisis","Relaspe","relapse","relapse (with ascites)")

then cause_of_death_grouped=**1**;

else if cause_death in ("Septic Shock", " Septic Shock","Aspergillosis","Aspergillus and CMV pneumonia, pulmonary GvHD","Aspergillus pneumonia",

"Aspiration pneumonia","BKV viremia/viruria","Bacterial Infection(st aureus bacteremia)","Bacterial infection, CNS toxicity",

"Bacterial infection, MDS relapse","Bacterial pneumonia, sepsis, adenovirus viremia","Bacterial sepsis","Bacterial sepsis, MOF","C.Diff colitis",

"Bacterial sepsis, influenza A, CMV viremia", "Bowel perforation- sepsis",

"CMV infections, E.coli pneumonia","CMV pneumonitis","CMV ventriculitis, fungal pneumonia (drop in counts due to CMV viremia)","CMV viremia",

"CMV viremia, BK Positive, Klebsiella UTI","CMV, BK infections, aGvHD gut","CMV-pneumonitis","Cellulitis lower limb, bacterial sepsis",

"Cerebral abscess/ sepsis","Dementia following HSV encephalitis","Diverticulitis","E. coli septic shock on a background of acute GVHD post DLI for relapsed PLL",

"Fungal infection","Fungal pneumonia, (drop in counts due to CMV viremia)","Gram Negative sepsis","H1N1 pneumonia with resp failure","HSV encephalitis",

"Hemophilus + aspergillus pneumonia","Hypoxemic respiratory failure, pneumonia, Massive spinal cord infarction, organ failure (CNS)","Infection",

"Interstitial pneumonitis, bacterial pneumonia","Mucor mycosis brain","Mucormycosis lung liver","Overlapping gut GvHD, E. coli sepsis, ITP",

"Progressive Multifocal Leucoencephalopathy due to JC Virus-viral infection",

"Protozoal Infection, MOF(brain, heart, kidneys)","Pseudomonas pneumonia", "Pseudomonas sepsis","Pseudomonas sepsis, BK cystitis","Pulmonary aspergillosis, cGvHD",

"RSV pneumonia","RSV, pseudomonal sepsis", "Resp Failure", "Resp. failure- viral pneumonia?","Respiratory Failure", "Respiratory failure",

"Respiratory failure Post lung transplant res","Respiratory failure, CMV infection","Respiratory failure, prev CMV",

"Respiratory failure, pulmonary infiltrates NYD","Respiratory failure- cause undetermined","Sepsis","Sepsis, GvHD","Sepsis, organism not identified",

"Sepsis, pneumonia","Septic shock", "Septic shock (pneumonia)", "Septic shock, febrile neutropenia, clostridium septicum",

"Septic shock- infection", "Severe C.difficile diarrhea",

"Staph aureus bacteremia tricuspid valve endocarditis acute lung injury CMV .pneumonia adenoviral pneumonia Resp failure, pneumothorax",

"Staph aureus septicemia, steroid refractory acute GVH","Stenotrophomonas, Aspergillosis, Nocardia","Strep viridans sepsis","Varicella encephalitis","Viral Infection",

"Viral Infection (Disseminated VZV, hepatitis, septic scock)","Viral encephalitis (?HHV6)","Viral infection","bacterial sepsis","gram neg sepsis",

"gram neg sepsis (pseudomonas on BAL) refractory septic shock with Multi Organ failure",

"hyoxemia secondary to recurrent aspiration pneumonia, bacterial, organ failure", "infection/pneumonia (organism not identified)",

"infections (e faecium, gram positive bacillus, candida), adenovirus, upper GI bleed and worsening lung consolidation","influenza, ITP","klebsiella sepsis",

"lung infection (organism not identified)","probably chest infection (local ER)","sepsis (unidentified organism)","sepsis, pulmonary fibrosis,",

"sepsis, query bacterial,fungal","severe pneumonia","viral infection (covid pneumonia)","Pneumonia","Pneumonia pseudomonas, CMV, yeast","Pneumonia, resp GvHD",

"Poor graft function due to CMV viremia, refractory CMV viremia")

then cause_of_death_grouped=**2**;

else if cause_death in ("GI GvHD steroid refractory", " GI GvHD steroid refractory","Acute GVH of gut","Acute GvHD GI, RBC fragmentation","Acute GvHD skin, GI",

"Acute GvHD skin, steroid refractory, infectious complications","AcuteGvHD liver and gut.","Chronic GVHD","Chronic GVHD, Non Identified Infection",

"Chronic GVHD; Liver failure","GVHD of gut","Grade IV Acute GVHD (Gut Stage 4, Skin 0, Liver 0); Sepsis; Multi Organ Failure","GvHD","GvHD gut",

"GvHD liver steroid refractory","GvHD skin, gut, liver","GvHD stage IV","Liver GvHD?","Recurrent liver cGvHD, cirrhosis","Refractory GvHD skin and gut",

"Refractory cGvHD","Refractory cGvHD liver","Refractory cGvHD multiple organs","Rejection failure - GVH of skin, liver",

"Steroid refractory GVH of the gut","Steroid refractory GvHD GI","Steroid refractory GvHD GI tract","Steroid refractory GvHD gut",

"Steroid refractory GvHD liver", "Steroid refractory aGvHD GI tract","Steroid refractory aGvHD gut and liver",

"Steroid refractory aGvHD liver, gut (following DLI)","Steroid resistant aGvHD gut","Steroid resistant cGvHD liver","Steroid-refractory GvHD gut",

"Steroid-refractory GvHD gut, liver","Steroid-refractory GvHD gut, relapse","Steroid-refractory GvHD liver","Steroid-refractory aGVHD gut",

"aGvHD GI and liver","acute GVH, multi factorial","acute GvHD","acute GvHD GI","acute GvHD GI","acute GvHD gut","acute GvHD skin, gut, liver (?) PTLD",

"acute on chronic GvHD, liver,skin,lung","cGvHD liver", "cGvHD liver, hepB reactivation, CMV","cGvHD lung","cGvHD, failure to thrive, BK","chronic cerebral GVH",

"severe chronic GVHD","steroid reffractory GvHD gut, liver, lung", "steroid refractory GVH of gut","steroid refractory GVHD",

"steroid refractory acute GVHD of gut", "steroid refractory acute GVHD of gut, liver", "steroid res GvHD, Mucor, CMV","steroid resitant GvHD GI tract",

"steroid resitant acute GvHD GI tract" )

then cause_of_death_grouped=**3**;

else if cause_death in ("AIHA","Bx proven VOD,","Cirrhosis, hemochromatosis","Graft failure","Guillian Barre syndrome",

"Head and Neck Ca, Invasive SCC esophagus, secondary malignancy","Hemopneumothorax","Hepato-renal Failure","Interstitial pneumonitis, bacterial pneumonia",

"Intracranial hemorrhage","Intracranial hemorrhage, (drop in counts due to CMV viremia)","Liver failuire (Cirrhosis)","MI following AAA surgery",

"MOF","Massive intracranial bleed following fall, intracranial hemorrhage, thrombocytopenia","Massive subdural hematoma","Multiple cerebral infarcts","PTLD",

"Pancreatic Ca","Pancytopenia due to TMA","Cardiac arrest,","Cardiac failure","Pulmonary embolism","Pulmonary embolism (?),","Second malignancy","Secondary Malignancy",

"Secondary malignancy-metastatic breast Ca","Suicide","Unknown","Unknown cause (cardiac?)","cardiac arrest","cardiac failure,","cerebral infarct",

"intracerebral hemorrhage after falling","liver failure, hypoxaemic respiratory failure","sudden death","Intracranial hemmorhage")

then cause_of_death_grouped=**4**;

**run**;

**data** opioid_user_no3; set opioid_user_no2; where cause_of_death__primary_ ne ""; keep cause_of_death__primary_; **run**;

**proc** **freq** data=opioid_user_no2;tables cause_of_death_grouped; **run**;

**proc** **freq** data=opioid_user_no2;tables cause_of_death__primary_; **run**;

**proc** **means** data=descriptive n mean std min median max; class user_final3; var days_to_discon_of_immuno; **run**;

**proc** **npar1way** data=descriptive wilcoxon; class user_final3; var days_to_discon_of_immuno; **run**;

/************ how many patients have overlapping prescriptions (more than one opioid prescription per day) *******************/

**data** opioid_user_yes_simple; set opioid_user_yes; keep study_ID;**run**;

**data** opioid6_simple; set opioid6; keep study_ID din_desc days_to_dt_of_serv_ts end_date; **run**;

**proc** **sql**;

create table overlap

as select * from opioid_user_yes_simple

left join opioid6_simple

on opioid_user_yes_simple.study_id=opioid6_simple.study_id;

**quit**;

/************************************************************************************/

/**************************** plot ************************************************/

/************************************************************************************/

**data** descriptive2;

set descriptive;

label user_final3="Opioid use";

**run**;

**data** cGVHD2;

set cGVHD;

label user_final3="Opioid use";

**run**;

/***** OS ******/

%***newsurv***(data=descriptive2, time=days_to_last_f_u3, cens=dead, cen_vl=**0**,summary=**0**,xincrement=**365**,EV_VL=**1**,display=legend hr pval,linesize=**6**, xmax=**1825**,

color=green red black blue, pattern=solid, class=user_final3,classref=Non opioid user, censormarkers=**0**,xlabel=Time (Days), ylabel=Overall Survival Probability (%),

title=Overall Survival by Opioid User (Yes vs No), plottype=EMF, svg=**1**, outdoc=X:\xxxxx\Output for Vetting\To output Nov **20**\Plot KM OS by Nov **20**);

/***** GRFS ******/

%***newsurv***(data=cGVHD2, time=GRFS_days, cens=GRFS, cen_vl=**0**,summary=**0**,xincrement=**365**,EV_VL=**1**,display=legend hr pval,linesize=**6**, xmax=**1825**,

color=green red black blue, pattern=solid, class=user_final3,classref=Non opioid user, censormarkers=**0**,xlabel=Time (Days), ylabel=GRFS (%),

title=KM of GRFS by Opioid User (Yes vs No), plottype=EMF, svg=**1**, outdoc=X:\xxxxx\Output for Vetting\To output Nov **20**\Plot KM GRFS by Nov **20**);

/***** RFS ******/

%***newsurv***(data=descriptive2, time=RFS_days, cens=RFS, cen_vl=**0**,summary=**0**,xincrement=**365**,EV_VL=**1**,display=legend hr pval,linesize=**6**, xmax=**1825**,

color=green red black blue, pattern=solid, class=user_final3,classref=Non opioid user, censormarkers=**0**,xlabel=Time (Days), ylabel=Relapse Free Survival Probability (%),

title=Relapse Free Survival by Opioid User (Yes vs No), plottype=EMF, svg=**1**, outdoc=X:\xxxxx\Output for Vetting\To output Nov **20**\Plot KM RFS by Nov **20**);

/***** NRM ******/

%***newsurv***(data=descriptive2, time=NRM_days, cens=NRM, cen_vl=**0**,summary=**0**,xincrement=**365**,EV_VL=**1**, method=CIF,display=legend hr pval,linesize=**6**, xmax=**1825**,

xlabel=Time (Days), ylabel=Cumulative Incidence of NRM (%),

color=green red black blue, pattern=solid, class=user_final3,classref=Non opioid user, censormarkers=**0**,

title=Cumulative Incidence of Non-Relapse Related Mortality by Opioid User (Yes vs No),

plottype=EMF, svg=**1**, outdoc=X:\xxxxx\Output for Vetting\To output Nov **20**\Plot CIF NRM by User Nov **20**);

/***** relapse ******/

%***newsurv***(data=descriptive2, time=relapse_days, cens=relapse_ind, cen_vl=**0**,summary=**0**,xincrement=**365**,EV_VL=**1**, method=CIF,display=legend hr pval,linesize=**6**, xmax=**1825**,

xlabel=Time (Days), ylabel=Cumulative Incidence of relapse (%),

color=green red black blue, pattern=solid, class=user_final3,classref=Non opioid user, censormarkers=**0**,

title=Cumulative Incidence of Relapse by Opioid User (Yes vs No),

plottype=EMF, svg=**1**, outdoc=X:\xxxxx\Output for Vetting\To output Nov **20**\Plot CIF Relapse by User Jan **17**);

**data** check;

set descriptive2;

time_to_relapse_check=relapse_days+begin_within_1yr_post2;

**run**;

**proc** **means** data=check median min max maxdec=**5**; class user_final3; var time_to_relapse_check; where relapse_ind=**1**; **run**;

**proc** **npar1way** data=check wilcoxon; class user_final3;var time_to_relapse_check; where relapse_ind=**1**; **run**;
