# Supplementary material for: Exploring the impact of opioid use on outcomes in allogeneic hematopoietic stem cell transplantation
Source: PLoS One. 2025 Apr 4;20(4):e0321073. doi: 10.1371/journal.pone.0321073 (PMC11970678; doi:10.1371/journal.pone.0321073)
Supplement: Attachment — Submitted filename: Response to Reviewers Opioids and alloBMT.docx [file pone.0321073.s003.docx]

**Response to Reviewers: Manuscript No. PONE-D-24-36417**

**Journal Requirements:**

**Comment 1:** Please ensure that your manuscript meets PLOS ONE's style requirements, including those for file naming.

**Reply:** We have reviewed the style requirements and ensured every file is named according to your guidelines.

**Response:** Headings and subheadings have been modified to meet PLOS ONE’s style requirements.

**Comment 2:** Thank you for stating the following in the Acknowledgments Section of your manuscript:

“This study contracted ICES Data & Analytic Services (DAS) and used de-identified data from the ICES Data Repository, which is managed by ICES with support from its funders and partners: Canada’s Strategy for Patient-Oriented Research (SPOR), the Ontario SPOR Support Unit, the Canadian Institutes of Health Research, and the Government of Ontario. This study was supported through provision of data by ICES and Cancer Care Ontario (CCO) and through funding support to ICES from an annual grant by the Ministry of Health (MOH) and the Ontario Institute for Cancer Research (OICR). The opinions, results, and conclusions reported in this paper are those of the authors. No endorsement by ICES or any of its funders or partners is intended or should be inferred.”

We note that you have provided funding information that is not currently declared in your Funding Statement. However, funding information should not appear in the Acknowledgments section or other areas of your manuscript. We will only publish funding information present in the Funding Statement section of the online submission form.

**Reply:** We greatly appreciate your thorough review and the guidance provided regarding the placement of funding acknowledgments within the manuscript.

**Response:** In response, we have made the following changes:

- **Funding Statement:** Updated to: “This study was supported through the provision of data by ICES and Cancer Care Ontario (CCO), with funding support to ICES from an annual grant by the Ministry of Health (MOH) and the Ontario Institute for Cancer Research (OICR). The authors did not receive any specific funding for this work.” Lines 438-441.
- **Acknowledgments Section:** Revised to: “This study contracted ICES Data & Analytic Services (DAS) and used de-identified data from the ICES Data Repository, which is managed by ICES with support from its funders and partners: Canada’s Strategy for Patient-Oriented Research (SPOR), the Ontario SPOR Support Unit, the Canadian Institutes of Health Research, and the Government of Ontario. The opinions, results, and conclusions reported in this paper are those of the authors. No endorsement by ICES or any of its funders or partners is intended or should be inferred.” Lines 426-432

**Comment 3:** We note that you have indicated that there are restrictions to data sharing for this study. For studies involving human research participant data or other sensitive data, we encourage authors to share de-identified or anonymized data. However, when data cannot be publicly shared for ethical reasons, we allow authors to make their datasets available upon request.

(a) If there are ethical or legal restrictions on sharing a de-identified dataset, please explain them in detail (e.g., data contain potentially identifying or sensitive patient information, data are owned by a third-party organization, etc.) and who has imposed them (e.g., a Research Ethics Committee or Institutional Review Board, etc.). Please also provide contact information for a data access committee, ethics committee, or other institutional body to which data requests may be sent.

(b) If there are no restrictions, please upload the minimal anonymized dataset necessary to replicate your study findings to a stable, public repository and provide us with the relevant URLs, DOIs, or accession numbers.

**Reply:** Thank you for your inquiry regarding the data-sharing policy from ICES.

(a) **Ethical and Legal Restrictions on Data Sharing:** The dataset used in this study is held securely in coded form at the Institute for Clinical Evaluative Sciences (ICES). Data-sharing agreements with ICES prohibit the dataset from being made publicly available due to ethical and legal restrictions. These restrictions ensure the privacy and confidentiality of the data, which include sensitive patient information. ICES operates under Ontario’s privacy legislation, which governs the collection, use, and disclosure of health data.

Access to the data can be granted to researchers who meet specific criteria for confidential access as outlined by ICES. Data access requests may be directed to ICES Data & Analytic Services (DAS) via their website: [www.ices.on.ca/DAS](http://www.ices.on.ca/DAS).

For additional information or data access requests, please contact: ICES Data & Analytic Services Email: das@ices.on.ca

(b) **Data Availability Statement Update:** Updated to: “The dataset from this study is held securely in coded form at ICES. While legal data-sharing agreements between ICES and data providers (e.g., healthcare organizations and government) prohibit ICES from making the dataset publicly available, access may be granted to those who meet pre-specified criteria for confidential access, available at [www.ices.on.ca/DAS](https://www.ices.on.ca/DAS) (email: das@ices.on.ca). The full dataset creation plan and underlying analytic code are available from the authors upon request, understanding that the computer programs may rely upon coding templates or macros that are unique to ICES and are therefore either inaccessible or may require modification to be used in other environments. Specific inquiries can be directed to the DAS team via email at [das@ices.on.ca](mailto:das@ices.on.ca).

Interested researchers can replicate our study findings in their entirety by obtaining access to the minimal dataset from ICES and following the methods outlined in our manuscript. The ICES DAS program provides a structured application process for researchers seeking access to ICES data, ensuring that all necessary conditions for data use are met. Additionally, the dataset creation plan and analytic code, available upon request, provide further guidance to enable replication.

We confirm that the authors did not have any special access privileges to the data. All researchers who meet ICES’s criteria for access and follow the application procedures can obtain the minimal dataset under the same conditions.

Unfortunately, uploading the complete minimal anonymized dataset to a stable, public repository is not possible due to the legal data-sharing agreements governing ICES data.” Lines 26-48.

**Comment 4:** Please ensure that you refer to Figure 2 in your text as, if accepted, production will need this reference to link the reader to the figure.

**Reply:** Thank you for your feedback.

**Response:** We have now referred to Figure 2 in the main text of the manuscript to ensure proper linkage and clarity for readers. Line 280.

**Review Comments to the Author**

**Reviewer #1:**

**Comment:** The manuscript was submitted with tracked changes and comments, making it very difficult and unpleasant to read. This feels more like a draft version than a completed manuscript.

**Reply:** Thank you for bringing this to our attention. We sincerely apologize for the oversight in submitting a version of the manuscript with tracked changes and comments.

**Response:** A clean, fully revised version of the manuscript, free of tracked changes and comments, has been uploaded for your review.

**Comment:** All predictors in Cox, Kaplan-Meier, and Grey's analysis must either be known at time 0 (transplant in this case), or be treated as time-dependent covariates (which is only available for Cox).

**Reply:** Thank you for raising this important point regarding the timing of predictors in our analyses. We would like to clarify our approach and explain how we addressed potential survival bias.

**Response:** The start time (time 0) for our analysis is not the time of transplant for all patients. As shown in Figure 2, we defined the start time as follows:

- For opioid users, the start time is defined as:
  - The first time opioids were used within one year post-BMT if the total duration exceeded 30 days.
  - The time of transplant if opioid use was initiated before BMT or within one year post-BMT for less than 30 days, but the patient died within one year while on opioids.
- The median start time for opioid users is 95 days post-transplant.

For the comparator group, the start time was adjusted to account for survival bias due to the delayed initiation of opioids in many patients in the opioid user group. Specifically:

- For patients who did not use opioids, the start time was set to time of BMT + 95 days.
- For patients who started opioids but did not meet the criteria for the opioid user group, the start time was set to time of opioid initiation + 95 days.

All predictors included in the Cox proportional hazards model, Kaplan-Meier analysis, and Grey's test were known at the defined start time (time 0). By aligning the start time for the comparator group with the median start time of the opioid user group (95 days), we accounted for survival bias, ensuring that both groups are analyzed from equivalent time points relative to their risk exposure. We hope this explanation addresses your concern. Please let us know if further clarification is needed. Lines 119-169.

**Comment:** The authors observe a correlation and not causation, but make claims toward the latter. The use of opioids may have actually followed a significant medical problem (for example, a relapse) and not the other way around. Therefore, the conclusions are unfounded.

**Reply:** Thank you for this insightful observation. We acknowledge that the use of opioids in our cohort could indeed have followed a significant medical event, such as a relapse, rather than being a contributing factor to it.

**Response:** That said, our study found that intense opioid use (IOU) was significantly associated with poorer outcomes, including a higher relapse rate at two years (31.4% vs. 16.4%, p=0.0049) and reduced relapse-free survival (29.4% vs. 52.5%, p<0.001). Furthermore, the median time to relapse was significantly shorter in the IOU group (147 days; range 52-393) compared to the non-IOU group (209 days; range 96-1793), with a p-value of 0.0082. Additionally, multivariate analysis indicated that IOU status was independently associated with a 2.32 times higher instantaneous rate of death compared to non-IOU (HR 2.32, 95% CI 1.5-3.5, p=0.002). These findings suggest that IOU status may serve as an important marker for identifying high-risk patients who could benefit from targeted interventions. Lines 300-301.

While we fully agree that correlation does not imply causation, we believe our findings provide an important basis for further exploration. The correlation observed raises critical questions about whether opioid use could have potential negative consequences in this vulnerable population, particularly given existing evidence of the immunosuppressive effects of opioids. It is not our intention to imply causation but rather to suggest that these results warrant further investigation through prospective studies to better understand the complex interplay between opioid use and outcomes in allogeneic stem cell transplantation patients. We have revised the manuscript to ensure that our language reflects the observational nature of the study and avoids any implication of causation. Lines 318-426.

**Reviewer #2:**

**Major Comments**

**Comment 1:** Title: Too strong, did we have any clue or proof how to explain this phenomenon beyond immunosuppressive effect of opiates.

**Reply:** Thank you for your observation regarding the title. As our analysis focuses on identifying correlations rather than proving causation, we understand the importance of ensuring that the title accurately reflects the study's observational nature.

**Response:** We have revised the title to: “Exploring the Impact of Opioid Use on Outcomes in Allogeneic Hematopoietic Stem Cell Transplantation.” This updated title better represents the exploratory and observational nature of the work while leaving room for future research to further investigate the mechanisms underlying the observed associations. Lines 1-2.

**Comment 2:** Aim: Since 30 years I am a clinical transplanter I hardly remember any patient of mine, except autologous myeloma patients, and patients with avascular necrosis or severe osteoporosis needs opiate prescriptions. I am not convinced why these patients are on opiates.

**Reply:** In North America, opioid prescriptions have historically been prevalent, particularly for managing moderate to severe pain in medically complex populations. For hematopoietic stem cell transplant (HSCT) patients, pain can stem from diverse sources, including mucositis, granulocyte-colony stimulating factor (G-CSF)-associated musculoskeletal pain, therapy-related neurotoxicity, and graft-versus-host disease (GVHD), among other complications. This clinical necessity is compounded by historical and systemic factors contributing to opioid prescribing trends, as highlighted by Alpert et al. (2022), including aggressive marketing and shifts in prescribing practices that significantly increased opioid use across patient populations, including those undergoing cancer and transplant treatments. Lines 87-88.

**Response:** As noted by Oh et al. (2020), effective pain management is essential for maintaining quality of life during the intensive treatment course of HSCT. Opioids, both short-acting and long-acting, are frequently utilized for addressing severe pain that is unresponsive to other interventions. The National Comprehensive Cancer Network (NCCN) guidelines underscore the importance of appropriate and safe opioid use in cancer-related pain management, particularly in cases of refractory or persistent pain. Moreover, Niscola et al. (2006) describe the dual nociceptive and neuropathic components of pain experienced by HSCT patients, further necessitating the use of opioids in certain cases.

In our cohort, the observed patterns of opioid use likely reflect these broader prescribing practices and the pressing clinical need to manage the multifaceted and often severe pain syndromes associated with HSCT. The disparity in prescribing practices between North America and other regions, as noted by Reviewer 2, was a key motivation for initiating this study. We aimed to investigate and contextualize these differences, which remain a significant concern given the implications for patient care and the ongoing opioid crisis.

**Comment 3:** Study population: Do patients start opiate use or the physicians? It is not clear in the paper. The paper excludes beforehand patients starting opiates after 1 year post-allo and underlines the bias for relapse vulnerable period. How do they know that opiates will have an impact on relapse beforehand? This is a hypothesis and should be performed in all opiate users, and selective analyses can be performed on selected populations.

**Reply:** Thank you for these clarifications.

**Response:**

- **Initiation of Opioid Use:** In our study, opioid use was initiated based on provider prescriptions in response to clinical indications such as moderate to severe pain related to post-transplant complications, including mucositis, musculoskeletal pain, or GVHD. This reflects standard clinical practice as outlined in pain management guidelines for hematologic malignancies and post-transplant care. We have revised the manuscript to make this clearer. Lines 236-238.
- **Exclusion of Patients Initiating Opioid Use Beyond One Year Post-Transplant:** The rationale for excluding patients who began opioid use more than one year post-transplant was based on our aim to study the impact of opioid use during the critical period of immune reconstitution and relapse vulnerability. Studies indicate that this period is characterized by significant risks for complications that could necessitate opioid use. However, we acknowledge that this criterion may introduce selection bias, as it limits the generalizability of our findings to early post-transplant opioid users. Lines 161-163.
- **Hypothesis Testing and Selective Analyses:** You are correct that the study is hypothesis-driven, exploring whether opioid use during this vulnerable period might correlate with an increased incidence of relapse. While our focus was on early opioid use, we agree that extending the analysis to all opioid users—regardless of the time of initiation—would provide a more comprehensive understanding of the relationship between opioid use and relapse. Selective analyses could then be performed on subpopulations, such as late-onset opioid users, to explore potential differences in outcomes. We will emphasize this limitation in the revised manuscript and highlight the need for future studies to include a broader range of opioid users. Lines 392-408.

**Comment 4:** Definition: According to which reference or infobase you established the definition of IOU. Why did you not include the non-malignant diseases? They suffer for a certain degree of transplant complications. The exclusion of benign disorders means that your intent is not only alloHCT-based but malignant hematology-based.

**Reply:** Thank you for your thoughtful comment regarding the definition of intense opioid use (IOU) and the exclusion of patients with non-malignant diseases. We appreciate the opportunity to clarify our rationale and approach.

**Response:**

- **Definition of Intense Opioid Use (IOU):** The definition of IOU in our study was based on previously published works, guidelines, and literature regarding opioid use in cancer-related pain management. Specifically, we referred to criteria from NCCN Clinical Practice Guidelines, which emphasize the clinical thresholds for opioid use in managing moderate to severe pain. This definition was tailored to identify patients with substantial and sustained opioid exposure to ensure consistency in our analyses.
- **Exclusion of Non-Malignant Disorders:** We excluded patients with non-malignant disorders to maintain a homogeneous study population. This decision was made because malignant and non-malignant diseases differ significantly in their pathophysiology, treatment courses, and associated complications. Including patients with benign conditions could introduce heterogeneity, potentially confounding the analysis of opioid use and its association with outcomes like relapse or survival in a malignancy-focused alloHCT setting.

While we acknowledge that patients with non-malignant diseases may experience transplant-related complications requiring opioids, our primary intent was to study the intersection of opioid use and malignant hematology within the context of alloHCT.

**Comment 5:** Methodology for relapse: For RFS we use a 2-year threshold but you did not consider patients starting to use opiates after 1 year; this approach is erratic.

**Reply:** Thank you for your thoughtful observation regarding the methodology for relapse-free survival (RFS) and our decision to exclude patients who initiated opioid use after one-year post-transplant. We appreciate the opportunity to provide further clarification.

**Response:**

- **Rationale for Exclusion of Patients Using Opioids After One Year:** The exclusion of patients who began using opioids more than one year post-transplant was implemented to minimize survival bias. Patients who survived beyond one year without requiring opioids may represent a distinct subgroup with inherently better outcomes. Including them could have introduced confounding factors that might obscure the relationship between early opioid use and relapse or survival outcomes.
- **Two-Year RFS Threshold:** While we used a two-year threshold for RFS, the exclusion criterion for opioid use after one year does not undermine the validity of our analysis within this framework. Rather, it ensures that we are studying patients with comparable exposure periods during the most vulnerable post-transplant phase. We acknowledge that this approach limits the generalizability of our findings to patients who begin opioid use later and have emphasized this limitation in the revised manuscript. Lines 410-421.

**Comment 6:** How do you biologically prove without knowing any exact dose, reason for prescription, etc., that intense use is correlated with poor outcomes? This is an observation of a retrospective single-center cohort, which has to be taken very cautiously.

**Reply:** Thank you for raising this critical point about the limitations of our study. We completely agree that our findings represent an observation of a retrospective, single-center cohort and that causation cannot be inferred from our results.

**Response:** Our study strictly reports a correlation between intense opioid use (IOU) and poorer outcomes in allogeneic stem cell transplantation (alloHCT) patients. We recognize that the retrospective nature of the study, lack of precise dosing data, and absence of information on the reasons for opioid prescription make it impossible to establish a causal relationship.

To address your concern, we now include additional data on the time to relapse. The median time to relapse was 147 days in the IOU group (range 52-393 days) and 209 days in the non-IOU group (range 96-1793 days), with a statistically significant difference (p=0.0082). This difference further highlights the need for future prospective studies to better understand the observed patterns. Line 300-301.

While our study does not provide direct biological evidence linking intense opioid use to poor outcomes, existing literature suggests potential mechanisms, such as the immunosuppressive effects of opioids, which could contribute to relapse or reduced survival. These mechanisms, while plausible, remain speculative in the context of our dataset and warrant further validation. Lines 367-391.

**Comment 7:** L409: "Given the widespread use of opioids in the alloHCT setting," is this correct? Do we have any clear data or publication so far since 60 years of alloHCT history?

**Reply:** Thank you for pointing this out. We appreciate the opportunity to clarify and substantiate this statement.

**Response:** There is indeed data in the literature highlighting the use of opioids in the alloHCT setting for managing transplant-related complications such as mucositis, graft-versus-host disease (GVHD), and other sources of severe pain. For example:

- Oh et al. (2020) evaluated the use of opioid analgesics in HSCT patients, demonstrating frequent and appropriate utilization for managing moderate to severe pain associated with post-transplant complications such as mucositis and musculoskeletal pain.
- Niscola et al. (2006) reviewed the role of opioids in managing pain in hematologic malignancies, emphasizing their necessity in addressing nociceptive and neuropathic pain often experienced in the post-transplant period.
- Geyer et al. (2020) highlighted that pain is observed in up to 60% of patients actively receiving treatment for hematologic malignancies and discussed safe opioid prescribing practices in this population.

We will include appropriate citations to ensure that the revised statement is well-supported and aligns with the available data. Lines 74-103.

**Minor Comments**

**Comment 1:** T1: Please define "Molecular" and "Cytogenetics" more clearly as a caption.

**Reply:** Thank you for pointing this out.

**Response:** We will revise the caption for Table 1 to provide a clearer definition of "Molecular" and "Cytogenetics" to enhance clarity and comprehension. Lines 249 and 251-252.

**Comment 2:** T2: Please revise as 2a UVA and 2b MVA. In its current form, it is not legible and difficult to evaluate.

**Reply:** We appreciate your feedback regarding the legibility of Table 2.

**Response:** To address this, we will separate the table into two parts for univariate analysis (UVA) and multivariate analysis (MVA) as "Table 2a" and "Table 2b." This format will improve clarity and ease of evaluation.

**Comment 3:** L380: "Several in vitro studies have shown that opioids can be immunosuppressive." This is very speculative. Either share details on the pathophysiology or revise.

**Reply:** Thank you for pointing out the need to elaborate on the mechanisms underlying the potential immunosuppressive properties of opioids.

**Response:** We have revised the discussion section to include a more detailed exploration of these mechanisms, as described in the literature. Lines 367-386.
